# Survivin Mediates Mitotic Onset in HeLa Cells Through Activation of the Cdk1-Cdc25B Axis

**DOI:** 10.21203/rs.3.rs-3949429/v1

**Published:** 2024-02-28

**Authors:** Pedro M. Cánovas

**Affiliations:** Department of Cancer Biology, University of Massachusetts, Medical School, Worcester, MA 01605

**Keywords:** Survivin, Cdk1, Cdc25B, mitosis, kinase, phosphatase, G2/M-phase checkpoint, mitotic catastrophe, cancer

## Abstract

The Survivin protein has roles in repairing incorrect microtubule-kinetochore attachments at prometaphase, and the faithful execution of cytokinesis, both as part of the *c**hromosomal* *p**assenger* *c**omplex* (CPC) (1). In this context, errors frequently lead to aneuploidy, polyploidy and cancer (1). Adding to these well-known roles of this protein, this paper now shows for the first time that Survivin is required for cancer cells to enter mitosis, and that, in its absence, HeLa cells accumulate at early prophase, or prior to reported before (2, 3). This early prophase blockage is demonstrated by the presence of an intact nuclear lamina and low Cdk1 activity (4). Importantly, escaping the arrest induced by Survivin abrogation leads to multiple mitotic defects, or *mitotic catastrophe*, and eventually cell death. Mechanistically, Cdk1 does not localize at the centrosome in the absence of Survivin pointing at an impairment in signaling through the Cdc25B-Cdk1 axis. In agreement, even though Survivin directly interacts with Cdc25B, both *in vitro* and *in vivo*, in its absence, an inactive cytosolic Cdc25B-Cdk1-Cyclin B1 complex accumulates. This flaw in Cdc25B activation can however be reversed in Survivin-depleted HeLa cell extracts to which the recombinant Survivin protein is added back. Finally, a role for Survivin in the Cdc25B-mediated activation of Cdk1 is confirmed by overriding the early prophase blockage induced in cells lacking Survivin through the expression of a gain-of-function Cdc25B mutant.

## INTRODUCTION

Sequential activation of the Cdk1 protein kinase is essential for correct entry of cells into mitosis (5). This process involves formation of a complex between Cdk1 and its activator Cyclin B1 (5), and removal of inhibitory phosphates on kinase residues Threonine 14 and Tyrosine 15 by the phosphatase Cdc25 (5). In this respect, Cdk1 can be activated by three Cdc25 isoforms (5), being Cdc25B the first one that acts on the kinase at the centrosome (6, 7), and the other two the ones that amplify the centrosomal signal in the cytosol and the nucleus (5).

Full activation of Cdk1 in the nucleus at late prophase is considered a *point-of-no-return* in mitosis (8). Here, the nuclear lamina is pulled apart as a result of Cdk1 phosphorylation of the Lamin B subunits, triggering disassembly of the nuclear membrane (4, 9, 10). Following this event, cytosolic microtubules are able to reach the kinetochores on the chromosomes, as cells transverse from late prophase to prometaphase (11, 12). Interestingly, this so-called *mitotic commitment*, which requires high levels of Cdk1 activity in the nucleus (13, 14), is a phenotype difficult to identify, and only a detailed microscopic and biochemical analysis of mitotic cells can differentiate between those that have irreversibly entered mitosis, and those that are not fully committed yet. In this context, a frail activation of Cdk1 often leads to *mitotic catastrophe* and cell death (15).

On a different aspect of mitosis, it is well-known the role of the Survivin protein in microtubule dynamics (1), and how, at this stage, Survivin malfunction leads to multiple mitotic defects (2, 3, 16, 17). Focussing on the role of Survivin at prometaphase, this protein is part of the CPC together with Borealin, INCENP and Aurora B, where it contributes to proper localization and activation of the complex at the centromere (1). Once at this location, Aurora B, the CPC’s catalytic subunit, can sense kinetochore tension, and help correcting faulty microtubule-kinetochore attachments in partnership with the microtubule depolymerase MCAK (1). Here, mistakes caused by CPC’s malfunction generally lead to a sustained spindle checkpoint that, when overridden, can cause chromosomes to mis-segregate giving rise to aneuploidy, a typical feature of many tumors (1).

Survivin has also been localized at the centrosome (18) but its role at this non-membranous organelle has not been fully elucidated yet. In this regard, Survivin depletion causes a *mini spindle* phenotype in *Xenopus* egg extracts (17) and HeLa cells (16, 19), which points at a failure in centrosome separation, an early mitotic event for which centrosomal Cdk1 activity is essential through its effect on Eg5 (20, 21). These data, and the fact that Survivin binds to both Cdk1 (22) and microtubules (17, 23), seems to suggest a role of Survivin in the proper function of the main mitotic kinase at the centrosome, which would placed the Survivin protein at the hub where decisions to initiate mitosis are made, however, challenge the current model, which envisions Survivin first acting at the spindle checkpoint (1, 2). Interestingly, despite the importance of this topic, and its possible implications in cancer development, the role of Survivin at an earlier time than prometaphase has not been studied. For this reason, with the objective to unravel the role of Survivin at mitotic onset in cancer cells, I decided to revisit the early mitotic events that follow abrogation of the Survivin protein in HeLa cells.

## RESULTS

### siRNA-Mediated Loss of Survivin Induces an Early Prophase Blockage

**Figure 1.**
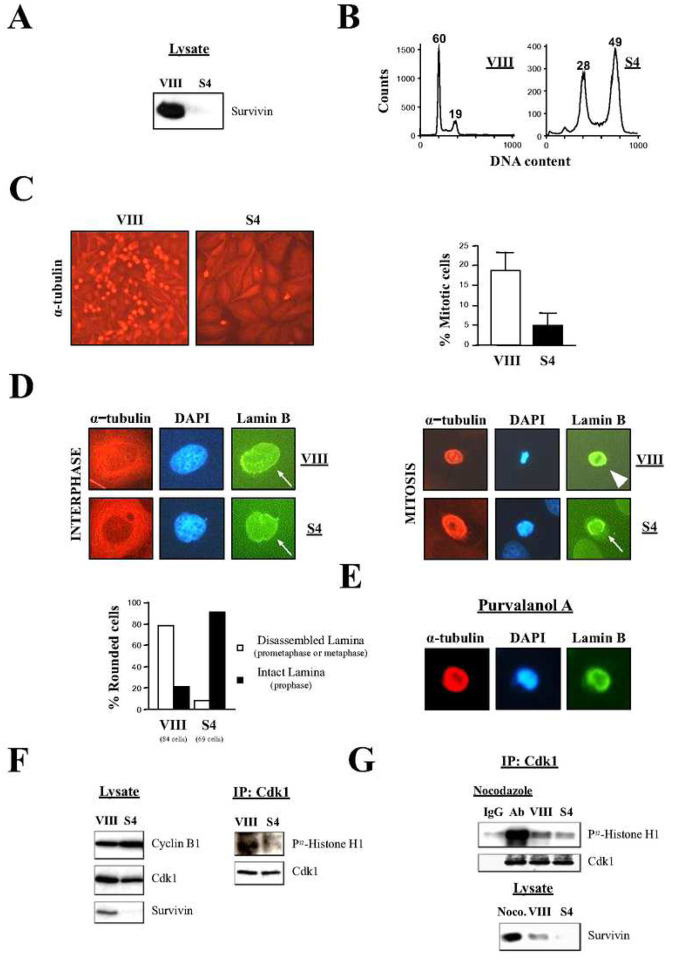
siRNA-mediated loss of Survivin induces an early prophase blockage in asynchronous HeLa cell cultures. *A, B*, Survivin knockdown. HeLa cells transfected with control (VIII) or Survivin (S4) siRNA were analyzed by Western blotting (*A*) or FACS analysis (*B*). The percentage of G1 (*2N*), G2/M-phase (*4N*) and polyploid (*>4N*) cells is indicated. *C*, siRNA-treated HeLa mitotic cells. HeLa cells transfected with the indicated siRNA were analyzed by fluorescence microscopy with an antibody to **α**-tubulin (left). Bar graph shows the percentage of mitotic cells in transfected cultures (n=4) (right). *D*, Nuclear lamina integrity in siRNA-treated HeLa cells. Interphase or mitotic HeLa cells transfected with the indicated siRNA, stained with an antibody to Lamin B or α-tubulin, and analyzed by fluorescence microscopy. DNA was stained with DAPI. Cells are shown at the same scale. Arrows show intact nuclear lamina, and arrow head points out at Lamin B colocalization with the mitotic spindle. The percentage of rounded mitotic cells with intact or disassembled nuclear lamina is indicated (*bottom*). Control (VIII) siRNA: 84 cells/8 fields; Survivin (S4) siRNA: 69 cells/12 fields (n=3). *E*, Purvalanol A treatment. HeLa cells were treated with the Cdk1 inhibitor purvalanol A (20 **μ**M), stained with an antibody to Lamin B or α-tubulin, and analyzed by fluorescence microscopy. DNA was stained with DAPI. F, Cdk1 kinase assay. siRNA-transfected HeLa cell lysates were used to immunoprecipitate (IP) Cdk1, and the kinase activity was analyzed by a Histone H1 phosphorylation assay (right). Amounts of Cyclin B1, Cdk1 and Survivin were analyzed by Western blotting as a control (left). *G*, Survivin ablation vs. prometaphase blockage. HeLa cells were treated with 10 μM nocodazole, and their Cdk1 activity was analyzed by an IP and a Histone H1 phosphorylation assay. Survivin siRNA-transfected HeLa cells were used as a control.

To start clarifying the stage at which cancer cells first arrest at mitosis following Survivin abrogation, I first repeated the RNAi experiments done by others in HeLa cells (16). As seen in Fig. 1A, treatment of asynchronous HeLa cell cultures with the Survivin siRNA (S4) caused a complete ablation of this protein, which correlated with an accumulation of cells at the G2/M-phase (28% vs. 19% in the control), and the detection of endoreplication (*>4N* DNA content) (49% vs. 0% in the control) by FACS analysis (Fig. 1B) (notice that in this particular experiment, no G1 cells (*2N* DNA content) could be seen in the absence of Survivin, indicating that no cells could complete mitosis).

Next, I wanted to visualize the mitotic stage at which the Survivin-depleted cells accumulating in the G2/M-phase peak (Fig. 1B) were at. To this aim, I treated asynchronous HeLa cell cultures with siRNA oligonucleotides as before, and labeled these samples with an antibody against the α-tubulin protein, so I could analyze them by immunofluorescence microscopy. As Fig. 1C, *left*, VIII panel shows, control cultures contained many mitotic cells, as judged by the large number of rounded (i.e. prophase through anaphase) and splitting (i.e. telophase/cytokinesis) cells with bright microtubules that could be detected. In contrast, HeLa cells where Survivin was ablated did not accumulate in mitosis, as concluded by the small number of rounded cells, and the absence of telophase figures seen under the microscope (Fig. 1C, *left*, S4 panel). Instead, in the absence of Survivin, I could observe many large multinucleated cells in agreement with the initial DNA content analysis (Fig. 1B). To accurately determine the extent of the defect in mitotic entry in the cells lacking Survivin, I counted the number of rounded cells from several microscope fields. As Fig. 1C, *right* shows, HeLa cell cultures where Survivin was ablated, contained about 30% less rounded cells than the control ones (5% vs. 18% in the control).

The drop in mitotic cells in cultures where Survivin was absent could also be seen in siRNA-transfected HeLa cultures analyzed by immunofluorescence microscopy using an antibody against the *bona fide* mitotic protein Cyclin B1 (Fig. S1). Here, I could observe that, following Survivin abrogation, most cells had a dispersed Cyclin B1 protein throughout the cytosol, a typical phenotype of late G2 or early prophase cells (24). Furthermore, the few Survivin-depleted cells that managed to enter mitosis appeared to be mostly in prophase, as judged by the colocalization of their Cyclin B1 protein and DNA (24). In contrast, Cyclin B1 colocalized with the spindle in the control samples, a typical feature of cells in prometaphase or metaphase (25). From these data, I concluded that abrogation of Survivin in HeLa cells leads to an impairment in their capacity to commit to mitosis, an event determined by the prophase to prometaphase transition, which is controlled by full Cdk1 activation (8).

In order to obtain better visual data on the Survivin-depleted mitotic cells, and in this way elucidate the phase at which the few cells that entered mitosis without Survivin were at, siRNA-treated rounded HeLa cells were further analyzed by immunofluorescence microscopy at higher resolution (Fig. 1D). Here, three cellular markers were used, namely: i) microtubule length, as shown by an α-tubulin antibody, where short bright microtubules indicate cells in mitosis, ii) chromatin condensation, observed by DAPI staining, where condensed chromatin signs mitosis cells from late prophase onwards, and iii) nuclear lamina integrity, as shown by a Lamin B antibody, which discriminates between interphase through prophase cells (i.e. intact nuclear lamina), and prometaphase and onwards (i.e. disassembled lamina) (4, 9, 10). As Fig. 1D, left panels show, staining of interphase cultures was the same, independently of the siRNA treatment, and consisted of cells with long microtubules and an intact nuclear lamina (arrows) encircling the chromatin. On the other hand, most control rounded mitotic cells assembled a spindle embracing condensed chromatin at the spindle equator, and had a disassembled nuclear lamina, a phenotype previously ascribed to nuclear Cdk1 activity (10), where the Lamin B monomers co-localized with the spindle (Fig. 1D, *right*, arrow head), a typical mitotic feature (26). In contrast, the few Survivin-knocked down rounded cells that could be observed had a partial condensed chromatin surrounded by layers of short microtubules that encircled the DNA, and an interphase-looking nuclear lamina (i.e. circular lamina), which also encircled the chromatin (Fig. 1D, *right*, bottom panels), pointing to these cells being at prophase. To determine the extent of this abnormal phenotype, a number of siRNA-treated rounded cells from several independent experiments were scored. As Fig. 1D, *bottom* shows, while 80% of the rounded cells treated with the control siRNA (VIII) had a disassembled lamina (i.e. cells in prometaphase and onwards), only 10% of the Survivin-depleted cells had a similar phenotype, reinforcing the idea of an impairment in Cdk1 activation and prophase to prometaphase transition in cells lacking Survivin.

If a role for Survivin in the activation of Cdk1 at prophase was real, HeLa cells treated with a Cdk1 inhibitor should also show the Lamin B ring detected when Survivin was absent. To test this possibility, HeLa cells were treated with the Cdk1 inhibitor purvalanol A, and analyzed by immunofluorescence microscopy as above. As Fig. 1E shows, the purvalanol A treatment replicated the intact-lamina and partially-condensed-chromatin phenotype seen in Survivin-depleted cells, further supporting a role for the Survivin protein in Cdk1 activation at early mitosis.

To obtain direct evidence of the Cdk1 function being regulated by the Survivin levels, I checked the kinase’s activity in the presence and absence of Survivin. To this aim, siRNA-transfected HeLa cultures were lysed, subjected to Cdk1 immunoprecipitation (IP), and their kinase activity was measured by a Histone H1 phosphorylation assay. As Fig. 1F, *right* shows, when Survivin was absent the activity of the Cdk1 protein was reduced in comparison to the control. Also, the same lysates did not show major differences in the amounts of Cdk1 and Cyclin B1 (Fig. 1F, *left*), demonstrating that the lower Cdk1 activity in the Survivin-depleted cells was not due to a reduction in the amount of the proteins contributing to the Cdk1-Cyclin B1 complex.

Together, the immunofluorescence and Cdk1 activity data contradicted previous findings, which claimed an accumulation of cells at prometaphase following Survivin abrogation (2, 3). To clearly show the difference between the blockage caused by Survivin abrogation, and a real high Cdk1 activity prometaphase arrest (27, 28), HeLa cells were either treated with siRNA oligonucleotides as before, or incubated with the microtubule-depolymerizing agent nocodazole, which causes a potent prometaphase blockage, and their Cdk1 activities were compared by using a Histone H1 phosphorylation assay. As Fig. 1G shows, HeLa cells lacking Survivin had a low Cdk1 activity, which contrasted with the potent Cdk1 activation observed when similar cells were incubated with nocodazole.

### The Early Prophase Blockage Caused by Survivin Abrogation in Asynchronous Cultures Can Be Replicated in Synchronized HeLa Cells, Precedes Endoreplication and Can Be Rescued by Exogenous Survivin

**Figure 2.**
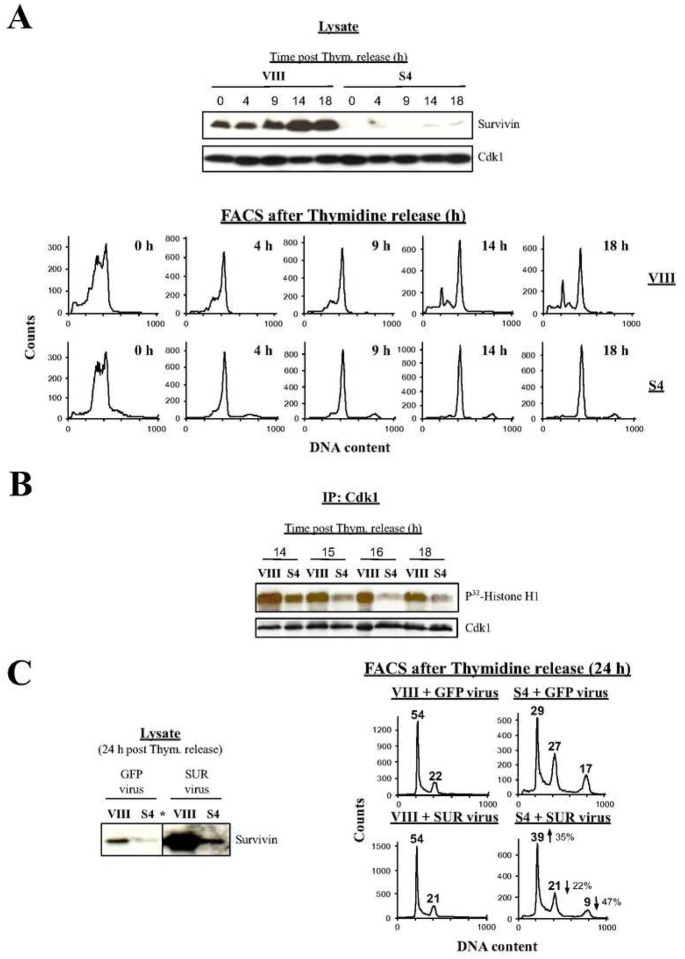
Survivin abrogation and reintroduction in synchronous HeLa cell cultures partially rescues the Survivin-induced blockage. *A*, Cell cycle analysis of siRNA-treated synchronized HeLa cells. HeLa cells transfected with control (VIII) or Survivin (S4) siRNA were synchronized with 2 mM thymidine for 48 h, released into fresh medium, and harvested at the indicated time intervals. Collected samples were subjected to Western blotting (*top*) or FACS analysis (*bottom). B*, Cdk1 activity during mitotic transition. Synchronized HeLa cells, previously transfected with the indicated siRNA, were harvested at the indicated time points following their release. Lysates were prepared and used to immunoprecipitate (IP) Cdk1, and the immune complexes were analyzed in a Histone H1 phosphorylation assay. C, Rescue of G2/M-phase blockage induced by Survivin ablation. Synchronized HeLa cells transfected with the indicated siRNA were transduced, following the thymidine release, with an adenovirus encoding GFP (pAd-GFP) or GFP-Survivin (pAd-Survivin), and subjected to FACS analysis after 24 h (*right*). The percentage of G1 (*2N*), G2/M-phase (*4N*) and polyploid (*>4N*) cells is shown. Arrows and percentages indicate changes in cell populations. Lysate panel shows expression of pAd-GFP or pAd-Survivin in siRNA-transfected HeLa cells (*left*) (*: Selected fields were cropped from the original blot and pasted next to each other (see Fig. S7A). Experiment was repeated two more times with similar results.

In order to pinpoint when the absence of Survivin first impairs mitotic progression, I repeated the above RNAi experiments this time using synchronous HeLa cell cultures. Fig. 2A, *bottom* shows the FACS analysis of control (VIII) or Survivin (S4) siRNA-transfected, synchronized HeLa cells (Fig. 2A, *top* shows the Survivin content in the synchronized transfected samples). As it can be seen, for the first 9 h following thymidine release, HeLa cells behaved exactly the same while transiting through interphase, and approaching the G2/M-phase peak, independently of the siRNA treatment, and only behaved slightly sluggish, probably as a result of the siRNA transfection (Fig. 2A, *bottom*). After this initial stage, I could see cells starting to diverge after 14 h following their release. Here, control cells initiated mitotic exit, and continued this trend for the remainder of the experiment (Fig. 2A, *bottom*, upper graphs), while Survivin-depleted cultures remained stuck, or minimally escaped the G2/M-phase blockage and endoreplicated (Fig. 2A, *bottom*, lower graphs).

To check whether the synchronous HeLa cells depleted of Survivin were also impaired in their ability to activate Cdk1 as their asynchronous counterparts, the Cdk1 activity of siRNA-transfected, synchronized HeLa cells was analyzed by a Histone H1 phosphorylation assay as before. Fig. 2B shows an immunoprecipitation (IP) of Cdk1 from lysates of control and Survivin-depleted cells, which were collected between 14 and 18 h post-thymidine release, or exactly when the control samples started exiting mitosis (see Fig. 2A, *bottom*, top graphs). As it can be seen, although the Survivin-knocked down cells initially mounted some Cdk1 activity, this could not be sustained during the time course and rapidly declined. In contrast, the phosphorylated Histone H1 signal was robust in the control samples, and started to decline at the end of the experiment following mitotic exit.

The data obtained with the synchronous cultures agreed with the asynchronous cell results, and supported a relationship between Survivin and Cdk activation on one hand, and mitotic progression on the other. If this theory were true, re-expression of Survivin in samples where this protein had previously been ablated should bypass the G2/M-phase blockage. To this end, siRNA-transfected, synchronized HeLa cells were incubated with a replication-deficient adenovirus encoding GFP (GFP virus) or GFP-Survivin (SUR virus) at the time of thymidine release, and lysates were analyzed by Western blotting (Fig. 2C, *left*) or FACS (Fig. 2C, *right*) 24 h later. As seen, lack of Survivin followed by transfection with GFP virus did not rescue the G2/M-phase cell cycle arrest (Fig. 2C, *right*, S4 + GFP virus). Notice here, the small G1 peak and the larger endoreplicated population in HeLa cells lacking Survivin as compared to Fig. 2A, *bottom*, lower graphs, which could routinely be seen in Survivin-depleted cells at very late time points (24 h) in the RNAi experiments. A plausible explanation for the G1 peak in the cells lacking Survivin might be some kind of recovery mediated by the small accumulation of Survivin at late time points (see Fig. 2C, *left*). On the other hand, the larger endoreplication peak could also be assigned to adaptation (i.e. *mitotic slippage*) at longer time courses. In contrast to the GFP adenovirus, expression of the SUR virus in Survivin (S4) siRNA-transfected, synchronous HeLa cultures reduced 47% the population of endoreplicated cells and 22% the G2/M-phase blockage, and increased 35% the population of cells that completed cytokinesis and entered G1 (Fig. 2C, *right*, S4 + SUR virus, arrows). Here, it should be noticed that the Survivin siRNA-treated cells transfected with the SUR virus showed a much lower adenoviral Survivin expression than the control cells, probably due to the still presence of the Survivin oligonucleotide in these samples, which certainly counteracted the effect of the exogenous Survivin protein on rescuing the G2/M-phase arrested Survivin-depleted cells.

### A Survivin Peptide Spanning Ala55 through Asp70 (SUR A55-D70) Binds the Cdk1 αC/β4 Loop

Before I started my studies, I was aware that Survivin and Cdk1 form a complex *in vivo* (22). Here, I expanded this result by showing that this interaction peaks as HeLa cells enter mitosis (Fig. S2A), and includes the Cyclin B1 protein (data not shown). To see whether the binding between Survivin and Cdk1 is direct, I expressed and purified GST-Survivin and His-Cdk1 from *E. coli*, and used these recombinant proteins in a GST-pull down experiment. As Fig. S2B shows, GST-Survivin but not GST pulled down the His-Cdk1 protein kinase, demonstrating that these two proteins bind directly. Due to this result, I could now address the question of whether Survivin is directly necessary for the activation of Cdk1 *in vivo* by making a peptide that could interfere with the cellular Survivin-Cdk1 complex. To this end, N- and C-terminal Survivin deletion mutants attached to a GST-tag were made, and used in GST-pull downs with His-Cdk1. Fig. S2C, *left* shows that His-Cdk1 interacted with the Survivin N-terminus (i.e. first 70 amino acids) but not the C-terminus (i.e. amino acids from 71 to 142). To narrow down the Cdk1-binding site in Survivin, more GST-Survivin deletion mutants were generated. As Fig. S2C, *right* shows, GST-pull downs using the new Survivin fragments showed that the minimal region in Survivin that binds to Cdk1 comprises the residues from Ala55 (A55) to Asp70 (D70), a region in the BIR domain with an abundant number of acidic amino acids (29) (Figs. S3A and C).

Following the identification of the Cdk1-binding Survivin region, I also wanted to know the region in Cdk1 that binds to Survivin. For this reason, I made several Cdk1 deletion mutants (Fig. S2D), which I used in binding assays with Survivin. Fig. S2D, *left* shows GST-Survivin pull downs using these kinase fragments. As seen, only the Cdk1 mutants His-Cdk1 1–183 and His-Cdk1 1–85, which contained the kinase N-terminal region, could bind to GST-Survivin but not GST. Next, I biotinylated the smallest Survivin region that binds to Cdk1 (SUR A55-D70), and used it in streptavidin-binding assays with all the His-Cdk1 fragments (Fig. S2D, *right*). As shown, all the Cdk1 mutants but His-Cdk1 182–297, His-Cdk1 1–56 and His-Cdk1 1–43 bound to SUR A55-D70. From these data, I concluded that the region in Cdk1 that binds to Survivin is located in the kinase N-terminal sequence, and comprises the amino acids from Lys56 (K56) to Met85 (M85), or a sequence that includes the αC/β4 loop of the kinase (Figs. S3B and D).

### The SUR A55-D70 Peptide Targets the Survivin-Cdk1 Complex, Inducing its Disassembly, Loss of Cdk1 Activity, Mitotic Abnormalities and Apoptosis

Before using the SUR A55-D70 peptide *in vivo*, I decided to carry out a few preliminary control experiments to assess its potential. First, I wanted to know whether the SUR A55-D70 peptide could pull down the Cdk1 protein from HeLa cell lysates. For this purpose, I incubated streptavidin beads pre-bound to the biotinylated scrambled or SUR A55-D70 peptide with HeLa cell extracts, and observed whether the endogenous Cdk1 protein bound or not to the resin. As Fig. S4A shows, endogenous Cdk1 bound to the beads that contained the Survivin peptide but not to the control reagent.

Second, I wanted to know whether the SUR A55-D70 peptide could displace the endogenous Survivin protein from its complex with Cdk1, and, if that was the case, whether this displacement affected the kinase activity. To this aim, I used a cell-free system, consisting of HeLa cell lysates from nocodazole-treated cultures, which should contain a large number of active Survivin-Cdk1 complexes. These cell extracts were supplemented with an ATP-regenerating system to make them biochemically active, and then incubated with the control or Survivin peptide. Fig. S4B shows that when the SUR A55-D70 peptide was incubated with nocodazole-treated HeLa cell lysates, and then Cdk1 was immunoprecipitated (IP), the kinase did not bind to the endogenous Survivin protein, and the Cdk1 activity was very low. In contrast, no effect on Survivin binding to Cdk1 or the kinase activity was observed with the control peptide.

Finally, before using the SUR A55-D70 peptide to analyze its *in vivo* effect, I wanted to know whether this reagent could transverse the HeLa cell membrane when attached to an N-terminal HIV tat cell-permeable sequence. Fig. S4C shows that both the scrambled and Survivin A55-D70 peptides attached to the HIV sequence, readily accumulated inside the cells following their incubation with HeLa cultures.

**Figure 3.**
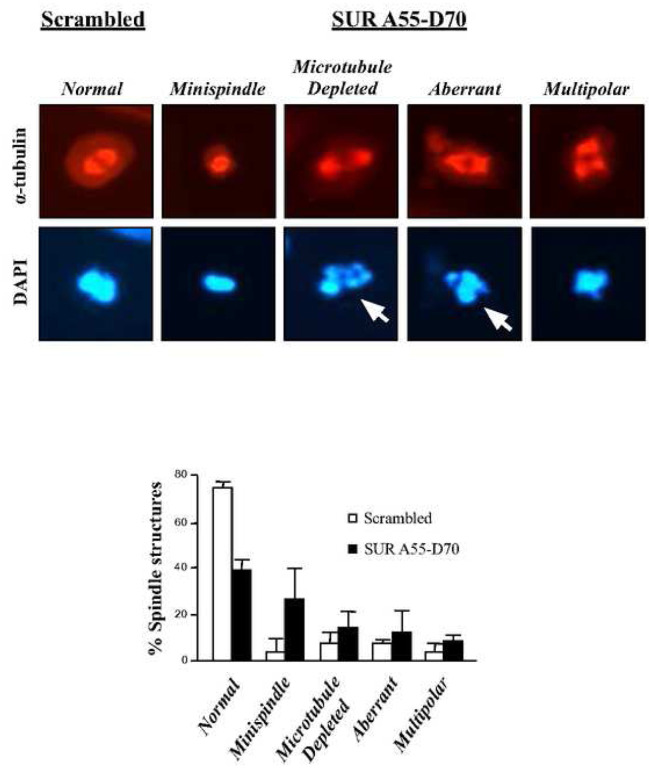
Treatment of HeLa cells with the SUR A55-D70 peptide causes spindle abnormalities. Spindle abnormalities caused by the SUR A55-D70 peptide. Asynchronous HeLa cell cultures were transfected with 50 μM scrambled or SUR A55-D70 peptide for 6 h, and cells were stained with an antibody to α-Tubulin. DNA was stained with DAPI (*top*). Mitotic phenotypes were quantified (*down*). Scrambled: 85 cells/10 fields; SUR A55-D70: 106 cells/12 fields (n=2).

Following the above controls, asynchronous HeLa cells were incubated with HIV tat-peptides (50 μM) for 6 h, and samples were analyzed by immunofluorescence using an α-tubulin antibody and DAPI staining. Here, I focused on rounded mitotic cells, as the Survivin-Cdk1 interaction that the Survivin peptide targeted peaked at this phase and correlated with a high Cdk1 activity (Figs. S2A and S4B). As Fig. 3, *top* shows, mitotic cells treated with the SUR A55-D70 peptide but not with the control reagent, showed several spindle abnormalities, which are summarized in Fig. 3, *bottom*. Here, it can be noticed that normal spindles were reduced by more than 50% in the mitotic cells treated with the Survivin peptide, as compared to the control. Also, this time I could see the *mini spindles* reported before (16, 17, 19). The *mini spindle* phenotype was 6 fold higher in the Survivin peptide-treated cells versus the control samples (28% vs. 5% in the control). Also, to a lesser extent, I could see other abnormal mitotic phenotypes with the SUR A55-D70 peptide, including microtubule-depleted, aberrant or multipolar spindles.

**Figure 4.**
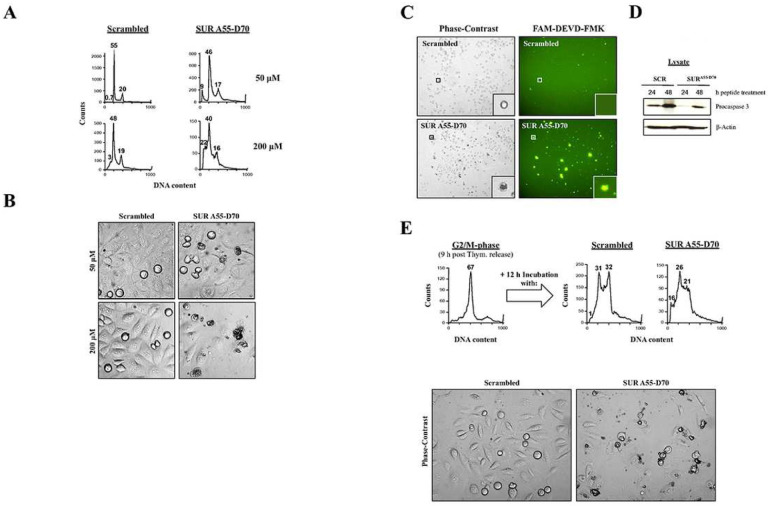
Prolonged incubation of HeLa cells with the SUR A55-D70 peptide causes apoptosis. *A*, *B*, SUR A55-D70-induced cell death. Asynchronous HeLa cell cultures were treated with 50 or 200 μM scrambled or SUR A55-D70 peptide for 24 h, and cells were analyzed by FACS (*A*) or phase-contrast microscopy (*B*). Images represent one of several experiments (n=4). The percentage of apoptotic (<*2N*), G1 (*2N*) and G2/M-phase (*4N*) cells is indicated in *A*. *C*, *D*, SUR A55-D70-induced apoptosis. Asynchronous HeLa cell cultures were transfected with 200 μM scrambled or SUR A55-D70 peptide, and cells were analyzed by phase-contrast microscopy and FAM-DEVD-FMK fluorescence microscopy (*C*) after 24h, or with an antibody to the the caspase 3 proform (*D*) after 24–48 h. Images represent one of several experiments (n=3). E, SUR A55-D70-induced apoptosis. G2/M-phase synchronized HeLa cells were transfected with 200 μM scrambled or SUR A55-D70 peptide for 12 h, and analyzed by FACS (*top*) or phase-contrast microscopy (*bottom*). The percentage of apoptotic (<*2N*), G1 (*2N*) and G2/M-phase (*4N*) cells is indicated.

When looking in detail at the DAPI staining of HeLa cells incubated with the SUR A55-D70 peptide, some chromosomes appeared to mis-segregate or be fragmented (Fig. 3, *top*, white arrows). This phenotype seemed to indicate some type of cell death, and was not observed before in the RNAi experiments. To investigate this possibility, I treated HeLa cells with the peptides as before, and checked their DNA content by FACS analysis after 24 h. Fig. 4A, top graphs show that when HeLa cells were incubated with 50 μM SUR A55-D70 peptide, a sub-G1 peak (9%) corresponding to cells with fragmented DNA (*<2N*) could be observed versus almost no effect in the control. Next, with the intention to enhance the cell death phenotype, I incubated HeLa cells with higher concentrations of the Survivin reagent, and again measured the amount of DNA by FACS analysis. Fig. 4A, bottom graphs show that the highest peptide concentration I could reach without causing too much toxicity in the control samples was 200 μM. Under these conditions the sub-G1 population was estimated to be 22% in the Survivin peptide-treated cells vs. 3% in the control.

To check if I could visualize the above sub-G1 population obtained after Survivin peptide treatment, I treated HeLa cell cultures with this reagent as before at either 50 or 200 μM concentration for 24 h, and analyzed these samples by phase-contrast microscopy. Fig. 4B shows that HeLa cells incubated with the Survivin peptide but not the control one, presented signs of cell death in a dose-dependent manner, which manifested as bubbles containing a dark condensed material, or cells that seemed to have bursted and spilled their contents out. Also, at the highest Survivin peptide concentration, I could see less cells under the microscope, suggesting a cytostatic or lytic effect of this reagent.

In order to determine the type of cell death observed in the HeLa cells transfected with the Survivin peptide, I treated HeLa cell cultures with 200 μM peptides as above, and assayed for caspase activity either by using the fluorescence probe FAM-DEVD-FMK, which labels the active caspase 3 and 7 enzymes in living cells (30), or incubating the samples with an antibody against procaspase 3, which cleavage indicates activation of the enzyme by caspases 8 and 9 (31). As Fig. 4C, left panels show, the same cell death phenotype as in Fig. 4B, right panels was observed in the Survivin peptide-treated cells but not in the control-treated samples. Also, when these images were compared to the FAM-DEVD-FMK labeling (Fig. 4C, right panels), the same cells, which had signs of cell death before showed activation of caspases 3 and 7 that could hardly be seen with the control reagent (Fig. 4C, *top*, right panel). Moreover, identically treated HeLa cell cultures as in Fig. 4C were used to analyze apoptosis using the procaspase 3 antibody. As Fig. 4D indicates, following the incubation of HeLa cells with the Survivin peptide for 24 or 48 h, a strong activation of caspase 3, as demonstrated by the disappearance of the caspase’s proform was seen in these samples, which was not detected in the control.

Since the Survivin peptide was targeting Survivin-Cdk1 complexes at mitosis, I reasoned that if I enriched for mitotic cells by using synchronous cultures, and then incubated these samples with the Survivin peptide, apoptosis might increase. To this aim, synchronous HeLa cells released for 9 h into fresh media (G2/M-phase) (Fig. 4E, *top*, left panel), were incubated with peptides as before for 12 h, and then analyzed by FACS analysis (Fig. 4E, *top*, right panels). As seen, in this particular experiment not all G2/M-phase cells treated with the control peptide had enough time to exit mitosis (31% G1 population), and some still remained in G2/M-phase (32%). However, the G2/M-phase to G1 transition in these samples was smooth, as concluded by the almost absence of cell death in these cultures (1%). In contrast to the control, the Survivin peptide-treated samples had a higher sub-G1 cell population (16%), which seemed to originate from the cells that stalled at G2/M-phase in the control, suggesting the possible better effectiveness of the Survivin peptide at causing cell death on populations transiting through mitosis.

Parallel peptide-treated, synchronous HeLa cell cultures as in Fig. 4E, *top* were analyzed under the phase-contrast microscope. As Fig. 4E, *bottom* shows, synchronized HeLa cells targeted with the SUR A55-D70 peptide at the G2/M-phase border died more readily than the asynchronous ones, as attested by the massive amount of apoptotic cells seen under these conditions, which interestingly was again higher than that observed in the FACS analysis. As with the asynchronous cultures, a lower number of cells could be counted in the Survivin peptide-treated samples, reiterating the possibility that this reagent might have cytostatic or lytic properties.

### A Survivin Asp70Ala/Asp71Ala (SUR D70A/D71A) Double Mutant Fails to Bind Cdk1 and Causes G2/M-Phase Arrest, Mitotic Abnormalities and Cell Death

To generate a second molecular antagonist of the cellular Survivin-Cdk1 complex, with which further investigate the role of Survivin at early mitosis, mutants of the Survivin domain spanning the Cdk1-binding region (i.e. amino acids Ala55 through Asp70) were generated by Alanine Scanning Mutagenesis, and screened for binding to recombinant His-Cdk1. As a result of this approach, several mutated polypeptides were identified that had a reduced binding capacity to Cdk1. These proteins included the single mutants SUR E63A, SUR E65A, SUR W67A and SUR D71A, and the double mutant SUR D70A/D71A (Fig. S5). From analysis of the mutant-binding assays, several things could be concluded. First, all the Survivin mutants, with the exception of SUR W67A, that did not bind to the Cdk1 kinase corresponded to mutations in acidic residues in the SUR A55-D70 region. Second, the best of these mutants was SUR D70A/D71A, and therefore this was the reagent chosen to be used in future studies. Finally, once the Survivin structure was revisited, it was hypothesized that the SUR D70A/D71A mutant might act as a dominant-negative protein since residues Asp70 and Asp71 appear on the surface of the Survivin dimer, and do not contribute to the monomers’ interface (32).

**Figure 5.**
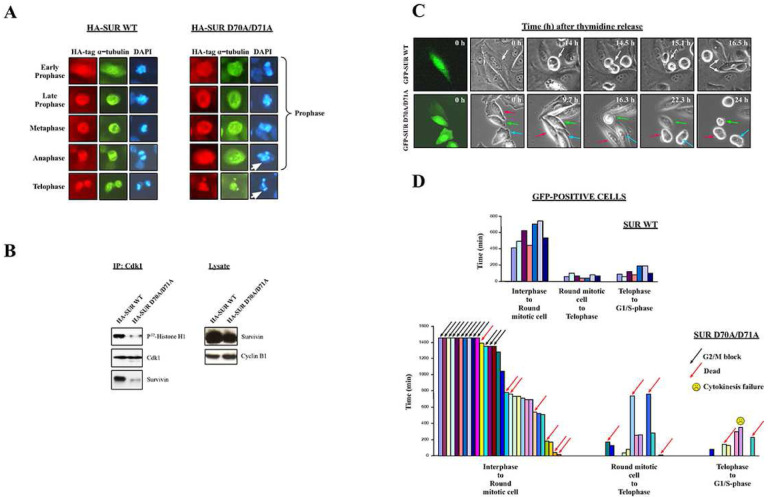
The Survivin Asp70Ala/Asp71Ala (SUR D70A/D71A) double mutant causes G2/M-phase arrest, mitotic abnormalities and cell death. *A*, SUR D70A/D71A causes mitotic abnormalities. Asynchronous Hela cell cultures were transfected with constructs expressing HA-tagged wild type Survivin (HA-SUR WT) or SUR D70A/D71A (HA-SUR D70A/D71A), and subjected to fluorescence microscopy using antibodies to HA-tag or α-tubulin (*top*). DNA was stained with DAPI. *B*, Cdk1 activity in SUR D70A/D71A-expressing HeLa cells. HA-SUR WT- or HA-SUR D70A/D71A-transfected HeLa cell lysates were used to immunoprecipitate (IP) Cdk1, and pellets were analyzed in a Histone H1 phosphorylation assay (*left*), or by Western blotting as a control (*right). C, D*, Time-lapse video microscopy of SUR D70A/D71A-expressing cells. Synchronous HeLa cell cultures were transfected with GFP-SUR WT or GFP-SUR D70A/D71A, released into fresh medium, and imaged every 10 min continuously for 24 h. Images were taken at the indicated time points. *C*, *bottom*, Selected cells undergoing sustained mitotic arrest (magenta and blue arrows), or cell death after mitosis re-entry (green arrow) are shown. *Top*, White arrow shows a control-transfected cell that progressed normally through mitosis. *D*, individual cells (bars), transfected with GFP-SUR WT (*top*) or GFP-SUR D70/D71A mutant (*bottom*), were quantified for time spent at each indicated mitotic transition and assigned specific phenotypes (color arrows).

Next, the SUR D70A/D71A protein was attached to a HA-tag and expressed in asynchronous HeLa cell cultures, which were analyzed by fluorescence microscopy. As shown in Fig. 5A, the most prominent mitotic abnormality observed here was a phenotype that resembled the cells in prophase, which were already reported with the Survivin siRNA (Fig. 1D). In effect, cells transfected with the HA-SUR D70A/D71A double mutant had microtubules that did not form clear spindles but instead surrounded their dispersed chromatin, and colocalized with the HA-tagged Survivin mutant protein (cells in Fig. 5A, right panels, labeled prophase). Also, this time some HA-SUR D70A/D71A-transfected HeLa cells appeared to have mis-segregated or fragmented DNA (Fig. 5A, white arrows), as some of the cells treated with the Survivin peptide. Coincidentally with the mitotic defects observed with the Survivin double mutant, HeLa cells expressing this protein also had a low Cdk1 activity, and a loss of binding of any Survivin protein to the Cdk1 kinase (Fig. 5B), confirming that it acted as a dominant-negative mutant.

In order to monitor mitotic progression in cells transfected with the Survivin double mutant, I synchronized HeLa cell cultures, followed by transfecting them with either the GFP-tagged wild type Survivin protein (GFP-SUR WT) or SUR D70A/D71A (GFP-SUR D70A/D71A) in the last 12 h of their thymidine treatment, and then released them into fresh media, so they could progress through mitosis with a fully expressed transfected protein. Here, two said experiments were carried out from which 7 control and 30 Survivin double mutant-transfected HeLa cells (green cells) were monitored by time-lapse video microscopy (Figs. 5C and D). As shown in these figures, synchronized control HeLa cells (Fig. 5C, top panels and Fig. 5D, top graph) rounded around 9.5 h, which corresponded to the G2/M-phase, as previously seen in the FACS analysis (see Fig. 2A, *bottom*, VIII graphs). These control cells remained rounded for an average of 74 minutes before initiating telophase/cytokinesis, a stage at which they stayed for an additional 121 minutes, as average, before spreading and entering G1.

Following a very different behavior (Fig. 5D, bottom graph), 13 out of the initial 30 GFP-SUR D70A/D71A-expressing HeLa cells remained flat (43%) for the 24 h that the time courses lasted. Of the other 17 green cells, 7 (23%) died early after their release into fresh media (3 cells in between 40–180 min), or later in the blockage (4 cells in between 540–1400 min or 9–24 h). The remaining 10 green cells, or 33% of the initial monitored population, rounded and entered mitosis with an average of 11.8 h or a 24% time delay in comparison with their control counterparts (average time 569 min). During mitosis, 4 out of the 10 cells that managed to escape the G2/M-phase blockage died before reaching telophase and cytokinesis (40%) (2 of them collapsing after 750 min or 12.5 h), and the other 6 progressed into the final mitotic stage, where they started splitting into two (half of these 6 cells after more than 250 min being round). Finally, during telophase/cytokinesis, 2 of the 6 cells died (33%), 1 did not complete cytokinesis, and only 3 managed to successfully complete mitosis, or 10% of the initial 30 cells used in the 2 time-lapse experiments.

A detailed microscopic analysis of individual HeLa cells expressing the Survivin double mutant showed some interesting behaviors. Representing these phenotypes, 3 cells labeled with a magenta, green or blue arrow are shown in Fig. 5A, bottom panels. Here, the cell labeled with the green arrow rounded very late (16.3 h), in agreement with the Fig. 5D, bottom graph, and then flattened up before dying after 24 h. To explain this behavior, I could only think that either this cell exited mitosis, and then died after trying to return to mitosis from G1, or alternatively, that it returned to G2, after prematurely entering mitosis, and then died when trying to re-enter prophase. Since, according to Fig. 5D, bottom graph, very few cells expressing the Survivin double mutant reached G1, I found the second explanation more plausible. Regarding the cells in Fig. 5C, bottom panels labeled with the magenta and blue arrows, they also rounded very late (22 h), and remained in this fashion for the rest of the time course. More interestingly, these cells had an elongated shape, similar to that seen in the RNAi experiments (Fig. 1D, bottom panels), which would be in agreement with deregulation of the Cdc25-Cdk1 axis (33).

### Survivin is Required for Recruitment and Activity of Cdk1 at the Centrosome

**Figure 6.**
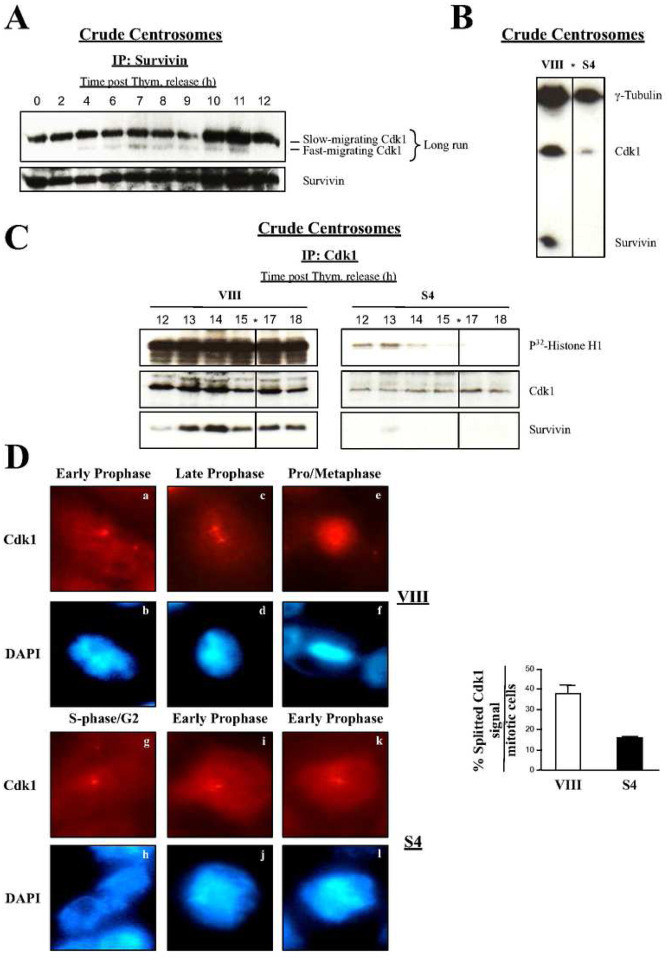
Survivin is needed to recruit Cdk1 to centrosomes. *A*, Fast-migrating centrosomal Survivin-Cdk1 complex at mitosis. Centrosomes were isolated from lysates of synchronous HeLa cell cultures, subjected to Cdk1 immunoprecipitation (IP), and analyzed by Western blotting. *B*, Centrosomal Cdk1 levels. Centrosomes were isolated from asynchronous HeLa cells transfected with control (VIII) or Survivin (S4) siRNA, and analyzed by Western blotting (*: Centrosome lanes were cropped from the original blot and pasted next to each other (see S7B). *C*, Centrosomal Cdk1 levels and kinase activity in siRNA-treated synchronous HeLa cultures. Centrosomal preparations from synchronized HeLa cells transfected with the indicated siRNA that were collected after release into fresh media at the mentioned times, were immunoprecipitated (IP) with an antibody to Cdk1, and pellets were analyzed by Western blotting or a Histone H1 phosphorylation assay (*: 16 h time point was omitted in both treatments due to a loading error). *D*, Centrosomal Cdk1 signal in Survivin-depleted cells. siRNA-treated HeLa cells were analyzed by fluorescence microscopy using an antibody to Cdk1 (*left*). DNA was stained with DAPI. Control cells (VIII) were either in prophase (a, b and c, d) or pro/metaphase (e, f), and Survivin-depleted cells (S4) were in S-phase/G2 (g, h) or early prophase (i-l). Percentage of splitted Cdk1 signal was scored (*right*) (n=3).

The above results pointed at a role of Survivin in the early activation of Cdk1. Activation of this kinase first occurs at the centrosome (6, 7), and interestingly, initial experiments with crude centrosomal preparations from synchronized HeLa cells, showed that when Survivin was immunoprecipitated (IP) from these samples, a fast-migrating Cdk1 band was bound (Fig. 6A) that was previously ascribed to the active kinase (27). The active centrosomal Cdk1 isoform first accumulated at 7 h, and preceded the FACS time point at which untreated HeLa cells started to come out of mitosis (8 h) (Fig. S2A, *right*). Furthermore, the Survivin-Cdk1 complex continued to be visible until 11 h, or right before all the synchronized HeLa cells reached G1 (12 h) (Fig. S2A, *right*).

To answer the question of whether Survivin is required for localization and/or activation of Cdk1 at the centrosome, I treated asynchronous HeLa cell cultures with siRNA oligonucleotides as before, and then prepared centrosomes, which were used to analyze their Cdk1 content by Western blotting. Fig. 6B shows that Survivin ablation caused a large decrease in the levels of the centrosomal Cdk1 protein, even after adjusting for γ-tubulin levels. To see if this result could be reproduced in synchronous HeLa cell cultures, samples were again transfected with siRNA oligonucleotides, synchronized, and aliquots were collected 12 to 18 h following the release into fresh media, a time that coincided with mitosis exit in the control cells (see Fig. 2A, *bottom*, upper panels). These samples were then used to prepare centrosomes from which Cdk1 was immunoprecipitated (IP), and the kinase activity measured. Fig. 6C, left panels show that a Survivin-Cdk1 complex accumulated and later slowly declined in the centrosome, which coincided with strong Cdk1 activity at this location. In contrast, much less Cdk1 was found and hardly any kinase activity was detected in the centrosomes of synchronized HeLa cells depleted of Survivin (Fig. 6C, right panels).

Centrosome dynamics is a well known process in eukaryotic cells (34). Briefly, in a G2 cell, the centrosome remains intact, although with duplicated centrioles. Following Cdk1 activation at prophase, the centrosome splits into two, and the daughter centrosomes gradually separate. Finally, as cells approach metaphase, the two spindle poles (i.e. daughter centrosomes) are fully separated, which coincides with the chromatin being totally condensed. Interestingly, when I started using my Cdk1 antibody in the early immunofluorescence microscopy experiments, I could identify one or two dots in mitotic HeLa cells, which seemed to mimic the above described centrosome dynamics during mitosis. This observation, I believed, could be used to identify the mitotic phase at which cells treated with different siRNAs were at. To this aim, I treated HeLa cells with siRNA oligonucleotides as before, and looked at them under the fluorescence microscope, following their labeling with the Cdk1 antibody (Fig. 6D, *left*). Here, I classified control cells transiting through mitosis in 3 categories: *a, b*, cells that contained a single Cdk1 dot and partially condensed chromatin, which I assigned to early prophase, *c, d*, cells with a partially separated Cdk1 signal, and uncompleted condensed chromatin, which I placed in late prophase, and iii) cells with two well separated Cdk1 dots, some spindle-localized Cdk1, and fully condensed chromatin, which I believed were at prometaphase or metaphase. In contrast to the control cells, a majority of the Survivin-depleted samples appeared to be in G2 (*g, h*) or early prophase (i-l), as indicated by their unseparated Cdk1 dot, and low chromatin condensation. To statistically determine the relevance of the unsplitted Cdk1 signal in cells depleted of Survivin, I counted this phenotype in several independent RNAi experiments. As shown in Fig. 6D, *right*, lack of Survivin caused a reduction of more than 2 fold (38% vs. 15%) in the number of cells with a splitted Cdk1 signal, supporting the role of Survivin in centrosomal dynamics and Cdk1 activation at this organelle.

### Recombinant Survivin Induces Cdc25 Activity *In Vitro*

**Figure 7.**
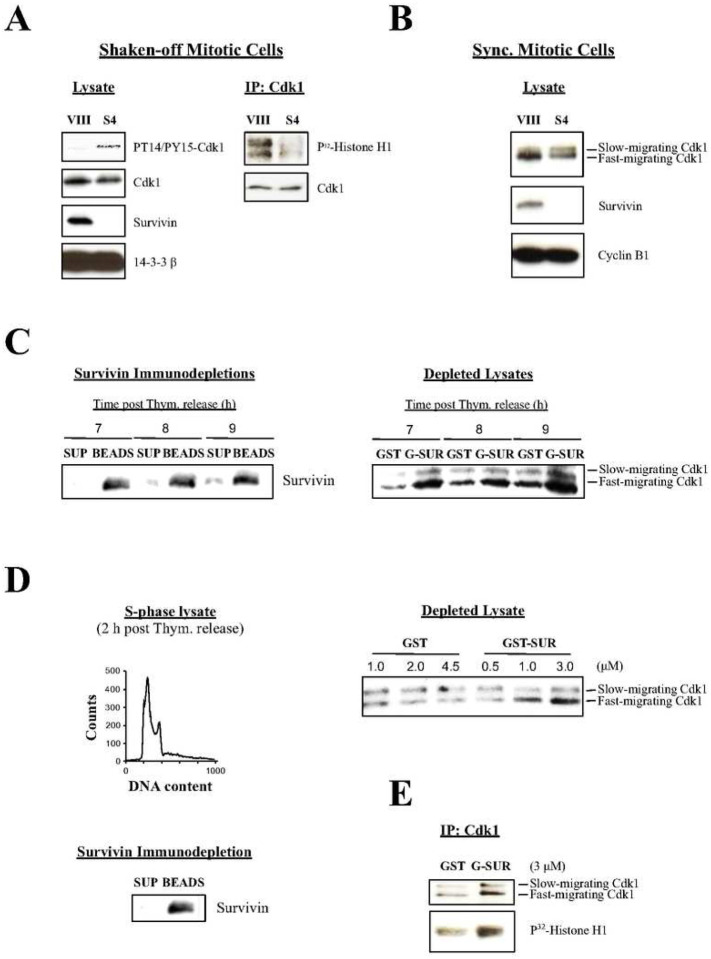
Cdc25 activity is induced by recombinant Survivin *in vitro*. *A*, Inactive Cdk1 isoform in Survivin-depleted cells. Shaken-off HeLa cells transfected with control (VIII) or Survivin (S4) siRNA were analyzed by Western blotting (left) or a Histone H1 phosphorylation assay (*right). B*, Impaired Cdc25 activity in Survivin-knocked down mitotic cells. Synchronized mitotic HeLa cells transfected with the indicated siRNA were collected, and analyzed by Western blotting. C, Induction of Cdc25 activity by recombinant Survivin *in vitro*. G2/M-phase HeLa cell lysates were depleted of Survivin (*left*), supplemented with an ATP-regenerating system, incubated with GST or GST-Survivin, and analyzed by Western blotting (*right). D, E*, Cdk1 activation by recombinant Survivin in interphase. *D*, Interphase HeLa cell lysates (*top left*), depleted of Survivin (*bottom left*), and supplemented with an ATP-regenerating system, were incubated with different concentrations of GST or GST-Survivin, and analyzed by Western blotting (*top right*), or a Histone H1 phosphorylation assay (*E*).

The above data indicated that Cdk1 does not localize at the centrosome following Survivin abrogation. Since Cdk1 is first activated by Cdc25B at this organelle (6, 7), it was tempting to speculate that Survivin might be bridging these two proteins at this location, and contributing to the initial Cdk1 activity. The mislocalization and accumulation of Cdk1 at the cytosol in the absence of Survivin would tip the balance towards the inactive kinase, which is phosphorylated at residues Thr 14 and Tyr 15 (PT14/PY15-Cdk1 protein) (27, 35) by the action of its negative regulators Wee1 and Myt1 (36, 37). To check this possibility, asynchronous HeLa cultures were treated with siRNA oligonucleotides as before, and lysates were prepared from shaken-off cells, which were analyzed by Western blotting using an antibody against the inactive Cdk1 form. As Fig. 7A, *left* shows, Survivin siRNA (S4) but not control (VIII) treated cells showed the inactive Cdk1 band. As a control, a Histone H1 phosphorylation assay of Cdk1 immunoprecipitated (IP) from the same samples was also performed to confirm that the PT14/PY15-Cdk1 band correlated with low Cdk1 activity (Fig. 7A, *right*).

As already introduced in the centrosomal studies, another way to differentiate between the inactive and active Cdk1 forms is by running proteins longer during SDS-PAGE (27, 35). To see whether I could use this approach to easily follow the state of Cdk1 activation, I used siRNA-treated synchronized cells as before that were released for 14 h (i.e. control cells exiting mitosis, see Fig. 2A, *bottom*), and lysates were prepared, which were subjected to a long SDS-PAGE run followed by Western blotting analysis using a Cdk1 antibody. As seen in Fig. 7B, a strong Cdk1 band was detected in the control cells. In contrast, in the Survivin siRNA-treated samples (S4), I could see a doublet, consisting of a faster migrating band, similar to the one in the control but fainter, and a slow-running Cdk1 form that should correspond to the phosphorylated inactive kinase (27, 35). From these and the results obtained with the PT14/PY15-Cdk1 antibody, I concluded that the lack of Cdk1 activity in the absence of Survivin was probably due to interference with the Cdc25-Cdk1 axis.

The above data could be more strongly supported if they could be replicated *in vitro*. To this aim, lysates from synchronized HeLa cells entering mitosis were prepared, which were used to deplete Survivin by incubating them with a Survivin antibody. The Survivin immunodepleted supernatants (Fig. 7C, *left*) were then incubated with either recombinant GST or GST-Survivin protein to see whether Survivin could induce the accumulation of the active Cdk1 form. As Fig. 7C, *right* shows, when Survivin-depleted HeLa cell lysates were incubated with GST, a small amount of the fast-migrating Cdk1 band could be seen that slightly increased from 7 to 9 h, as should be expected in cells approaching mitosis. In contrast, Survivin-depleted HeLa lysates that were supplemented with GST-Survivin clearly showed a higher amount of the fast-migrating Cdk1 protein at all times versus the control, which also significantly increased throughout the time course.

Next, I performed a titration experiment to see whether the accumulation of the fast-migrating Cdk1 band was dose dependent. This time, I used S-phase extracts, which should have almost no active Cdk1, and therefore provide a cleaner background. Here, it was also interesting to test whether Survivin could induce the activation of Cdk1 in interphase. This approach however had a caveat, which was that, under these conditions, Cdk1 might not be activated at all due to low upstream positive signaling in the interphase samples. To check these possibilities, Survivin-depleted HeLa cell lysates prepared from synchronized cultures collected 2 h post thymidine release (Fig. 7D, *left*) were incubated with increasing amounts of GST-Survivin or GST, and accumulation of the fast-migrating Cdk1 band was monitored. As seen in Fig. 7D, *right*, increasing amounts of GST-Survivin but not GST induced the accumulation of the fast-migrating Cdk1 band in a dose-dependent manner. Finally, to see whether this S-phase, fast-migrating Cdk1 isoform was active, a Histone H1 phosphorylation assay was carried out. As expected, Fig. 7E shows that, even in interphase, where Cdk1 and Cdc25 activities should be low, recombinant Survivin could mount some activation of the mitotic kinase.

### Survivin is Involved in the Activation of the Cdc25B Phosphatase

**Figure 8.**
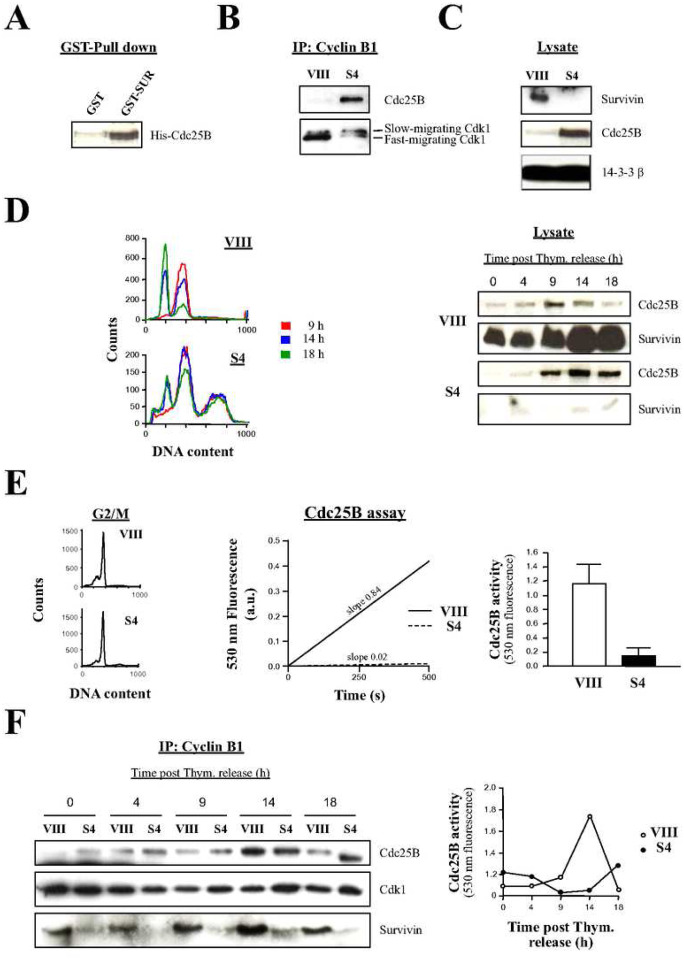
Survivin regulation of Cdc25B phosphatase activity. *A*, Survivin binds directly to Cdc25B. GST or GST-Survivin was mixed with His-Cdc25B, and analyzed by Western blotting. *B*, Accumulation of a cytosolic Cdc25B-Cdk1-Cyclin B1 complex in the absence of Survivin. Asynchronized HeLa cells transfected with control (VIII) or Survivin (S4) siRNA were collected, lysates were used to immunoprecipitate (IP) Cyclin B1, and pellets were analyzed by Western blotting. C, Cdc25B accumulation in asynchronous Survivin-depleted HeLa cell cultures. HeLa cells treated with the indicated siRNA were analyzed by Western blotting. *D*, Cdc25B accumulation in synchronous cell cultures depleted of Survivin. siRNA-treated synchronous HeLa cell cultures were analyzed by FACS (*left*) or Western blotting (*right*). *E*, Cdc25B activity at mitosis onset in siRNA-treated HeLa cells. Synchronized HeLa cells transfected with the indicated siRNA were collected at G2/M-phase (*left*), and used to immunoprecipitate (IP) Cdc25B. Cdc25B phosphatase activity in the immune complexes was measured by OMFP hydrolysis (*middle*). The result of several of these experiments (n=4) is shown on the *right. F*, Cell cycle Cdc25B activity in siRNA-treated HeLa cells. Synchronized HeLa cells transfected with the indicated siRNA were collected at the indicated times following thymidine release, and used to immunoprecipitate (IP) Cyclin B1. Immune complexes were analyzed by Western blotting (*left*). Cdc25B was immunoprecipitated from the same samples and its activity measured by OMFP hydrolysis (*right*).

As mentioned above, an explanation for the Survivin-mediated activation of Cdk1 in early prophase might be its mediation in the formation of a Cdc25B-Cdk1 complex at the centrosome. Accordingly, and since earlier in this paper, the direct interaction between Survivin and Cdk1 (22) was confirmed (Fig. S2B), I also wanted to know whether Survivin could directly bind to Cdc25B. Fig. 8A shows that when GST-Survivin was incubated with recombinant His-Cdc25B, these two proteins bound to each other. From this result, I was confident that I would find an *in vivo* Cdc25B-Cdk1 complex in the cells expressing Survivin. To my surprise, however, when I immunoprecipitated (IP) the Cdk1-Cyclin B1 complex from siRNA-transfected HeLa cells using a Cyclin B1 antibody, I could not see Cdc25B attached to this complex in the control samples but counterintuitively only in the cells that lacked Survivin (Fig. 8B). The Cdc25B-Cdk1-Cyclin B1 complex correlated with a low intensity, fast-migrating Cdk1 band, or active kinase, and the slow-moving Cdk1 form previously ascribed to the inactive kinase (27, 35). The Cdc25B-Cdk1-Cyclin B1 complex had to be cytosolic, as almost no Cdk1 protein was found in the centrosomes of the Survivin-depleted samples (Figs. 6B and C). Interestingly, the difference in the amount of Cdc25B bound to Cdk1-Cyclin B1 in the Survivin siRNA-treated samples replicated the amount of the total phosphatase in the cell lysates (Fig. 8C).

Next, I decided to study the dynamics of the Cdc25B protein in synchronous cultures. To this aim, siRNA-treated HeLa cells were synchronized as before, released into fresh media, and their amount of Cdc25B protein was monitored as they progressed into mitosis (Fig. 8D). As it can be seen, the amount of Cdc25B increased as control cells approached the G2/M-phase checkpoint, and then declined as cells exited mitosis (Fig. 8D, *left*). In contrast, in the Survivin-depleted samples, Cdc25B reached similar levels as in the control at the G2/M-phase but then its amount was sustained for the remainder of the time course, replicating the accumulation of this protein in asynchronous cultures (Fig. 8C). Here, unexpectedly, the FACS analysis data (Fig. 8D, *left*) showed that some Survivin-depleted cells managed to escape the G2/M-phase blockage. However, this could be explained as a small amount of Survivin could be seen after 14–18 h (Fig. 8D, *right*).

The Cdc25B-Cdk1-Cyclin B1 complex, and specifically the Cdc25B protein bound to it, which was detected in the asynchronous HeLa cells seemed to be inactive (Fig. 8B), and indeed had to be in order to agree with the Histone H1 phosphorylation assays (Figs. 1F and 7A). To see if this was true, I decided to immunoprecipitate (IP) Cdc25B from siRNA-treated synchronous cells entering mitosis (Fig. 8E, *left*), and check their phosphatase activity by employing the artificial substrate 3-O-methylfluorescein phosphate (OMFP) (38). As seen in Fig. 8E, *middle*, when the Cdc25B activity of Survivin-depleted cells about to enter mitosis was measured, a residual phosphatase activity was detected (0.18 a.u.), which contrasted with the strong activation of the phosphatase in the control samples (1.2 a.u.), or a 6.7 fold difference. This experiment was repeated four times and the results were very reproducible as shown in Fig. 8E, *right*.

To expand the knowledge on the Cdc25B-Cdk1 complex formation, and the activation of the Cdc25B phosphatase throughout the cell cycle, siRNA-treated samples were prepared as before, and their Cdk1-Cyclin B1 complexes were immunoprecipitated (IP) by using a Cyclin B1 antibody (Fig. 8F, *left*). Alternatively, Cdc25B was immunoprecipitated and used to measure its activity by OMFP hydrolysis (Fig. 8F, *right*). As Fig. 8F, *left* shows, Cdc25B formed a transient complex with Cdk1 and Cyclin B1 in control HeLa cells, which correlated with maximal phosphatase activity (14 h) (Fig. 8F, *right*), and mitotic exit (Fig. 8D, *left*). In contrast, in the absence of Survivin, Cdc25B remained bound to Cdk1 and Cyclin B1 from 4 to 18 h (Fig. 8F, *left*), and the activity of the phosphatase was negligible during all time points tested (Fig. 8F, *right*).

### Only a Dominant-Positive Cdc25B Mutant Can Bypass the Early Prophase Blockage Caused by Survivin Abrogation

**Figure 9.**
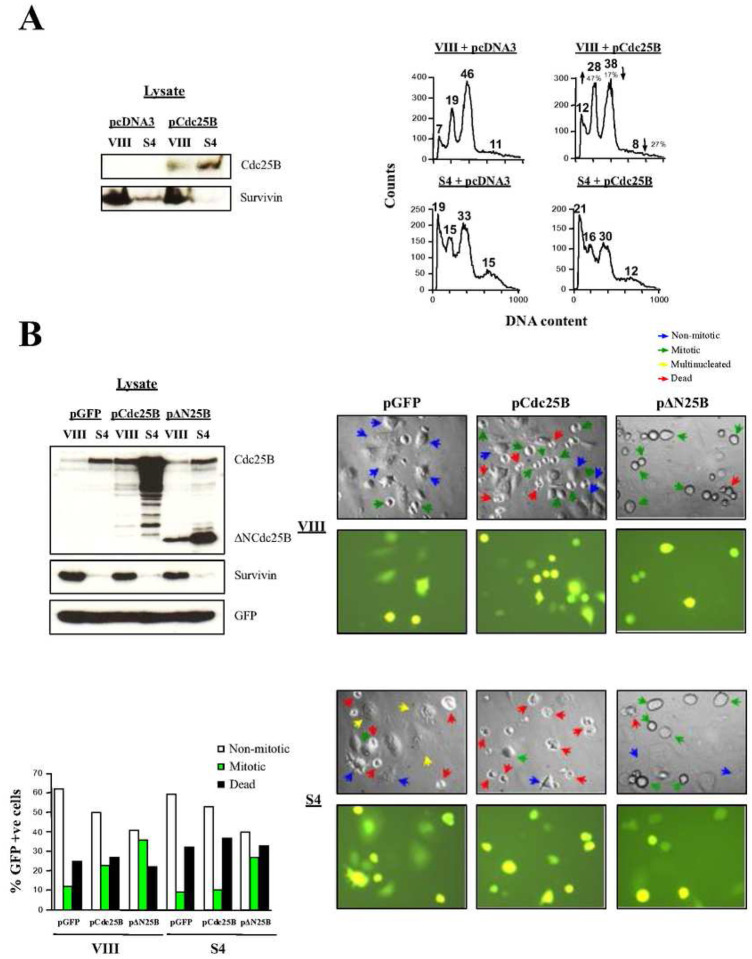
A gain-of-function Cdc25B mutant can override the blockage induced by Survivin abrogation. *A*, Cdc25B-mediated mitotic entry. HeLa cells transfected with control (VIII) or Survivin (S4) siRNA were synchronized, and then transfected with a pcDNA3 or pCdc25B plasmid, released and collected at 16 h. Cells were analyzed by Western blotting (left) and FACS (*right*). The percentage of cells with apoptotic (*<2N*), G1 (*2N*), G2/M-phase (*4N*) and polyploid (*>4N*) cells is indicated. Arrows and percentages indicate changes in cell populations. B, Gain-of-function ΔNCdc25B mutant overrides blockage induced by Survivin abrogation. siRNA-treated synchronized HeLa cells were transfected with a pGFP (control), pCdc25B (wild type Cdc25B) or pΔNCdc25B (constitutively active Cdc25B mutant) plasmid (the last 2 were also transfected with the GFP monitoring plasmid), released into fresh medium, and analyzed by Western blotting (*left*), or fluorescence and phase-contrast microscopy (*right*). Experiment was repeated 5 times with similar results. Phenotypes for one single experiment are quantified in *bottom left*.

To confirm the effect of Survivin abrogation on Cdc25B activity, the potent capacity of the phosphatase Cdc25B to induce mitotic entry when transfected into HeLa cultures (39) could be tested. Here, if Survivin had a role in Cdc25B activation, overexpression of the phosphatase in Survivin-depleted cells would not induce entry into mitosis. To this end, the FACS profiles of siRNA-treated, synchronized HeLa cells transfected with a Cdc25B construct were analyzed at mitotic exit (Fig. 9A). Fig. 9A, *right*, upper graphs show that Cdc25B overexpression in control samples resulted in an increase in the number of cells that progressed through mitosis (28% vs. 19%, or a 47% increase), as indicated by the larger G1 peak in comparison with the control (see arrows). In contrast, Survivin-ablated, synchronized HeLa cells transfected with the Cdc25B plasmid remained unaltered at G2/M-phase (Fig. 9A, *right*, lower graphs), as indicated by their almost unchanged G1 peak (16% vs. 15%, or 6.7% difference).

Because Survivin appeared to be indispensable to activate the Cdc25B phosphatase, and subsequently Cdk1, I predicted that a constitutively active Cdc25B mutant being expressed in the Survivin-ablated cells would bypass the G2/M-phase blockage. To this end, a construct encoding the truncated Cdc25B protein, containing its catalytic domain but not its N-terminal regulatory region (ΔNCdc25B) was generated as before (40), and transfected together with a GFP-monitoring plasmid in siRNA-treated cells (a full-length Cdc25B construct was also used as a control), and efficient transfection was detected by fluorescence microscopy. Simultaneously, I checked that the recombinant proteins were correctly expressed. As Fig. 9B, *left* shows, lysates of siRNA-treated HeLa cells transfected with the pCdc25B or pΔNCdc25B constructs showed accumulation of these proteins. Here, an interesting pattern could be noticed, which was that both the exogenously-expressed full-length and truncated Cdc25B phosphatases accumulated to a large extent in the cells lacking Survivin, and followed the behavior of the cells only expressing the endogenous phosphatase under the same conditions. Individual synchronized HeLa cells transfected with the control (VIII) or Survivin (S4) oligonucleotide, and expressing the recombinant Cdc25B proteins are shown in Fig. 9B, *right*. Here, it can be seen that control (VIII) siRNA-treated HeLa cells that were transfected with pCdc25B showed more mitotic cells than cultures, which were transfected with pGFP alone, in agreement with the data in Fig. 9A, *right*. In contrast, Survivin-knocked down cells transfected with pCdc25B did not enter mitosis when compared to the control but, on the contrary, died more readily. On the other hand, both HeLa cells treated with control (VIII) or Survivin (S4) siRNA, followed by transfection of the constitutively-active Cdc25B mutant, clearly entered mitosis when compared to their respective controls. One representative experiment of this sort appears quantified in Fig. 9B, *bottom left*. Here, it can be seen that ΔNcdc25B was effective in bypassing the G2/M-phase blockage associated with Survivin abrogation, and concomitant low Cdk1 activity reported in these studies.

## DISCUSSION

Up to date, the Survivin protein has only been implicated in the regulation of the spindle checkpoint and cytokinesis, both functions as part of the CPC (1). In agreement with these roles, it was firmly established that Survivin abrogation first leads to a prometaphase blockage, as a consequence of errors in the correction of faulty microtubule-kinetochore attachments (2, 3).

Before this project started, I was aware of experiments carried out with *Xenopus* egg extracts (17) and HeLa cells (16, 19), which revealed that, following Survivin abrogation, a distinctive phenotype was observed, consisting of spindles with a very short pole-to-pole distance, or *mini spindles*. Here, I believed that these results could poorly be explained by the sole role of Survivin in the CPC. A more plausible explanation might be a function of Survivin in centrosome separation, an event for which Cdk1 is necessary (20, 21). In this respect, it is also known that one of the consequences of interfering with the Survivin function is the detection of centrosomal abnormalities, and the disruption of a survivin-caspase-3-p21 complex at this location (18). Trying to connect the above data, I believed that if Survivin had a role in Cdk1 activation at the centrosome, this would place this protein at the hub where decisions to enter mitosis are made, and might explain Survivin connection to cell death as a consequence of the activation of a default centrosomal-centered apoptotic pathway (18) when mitotic onset is deregulated. This possible new role of Survivin in mitosis, however interesting, had never been studied before, and therefore, I intended to make it the center of my work.

### Survivin Is Required to Activate Cdk1 in Early Prophase and for Cancer Cells to Commit to Mitosis

When I started this project, I first wanted to clarify when cells that lack Survivin first block in mitosis. For this purpose, I decided to repeat the experiments carried out by others using RNAi in HeLa cells, which led these authors to conclude that in the absence of Survivin a prometaphase blockage ensues (2, 3). To my surprise, when I repeated this work, I could not see many cells blocked at prometaphase but cells that had an intact nuclear lamina and low Cdk1 activity, both phenotypes indicative of cells arrested at prophase. If this was confirmed, it would mean that Survivin had a new role upstream of its CPC function, which, when interfered with, would lead to nuclear Cdk1 inactivation (4, 9, 10), and subsequent failure at the prophase/prometaphase transition, impairing *mitotic commitment* (13, 14). The validity of the abnormal lamina phenotype and its connection to low Cdk1 activity was corroborated by treating HeLa cells with the Cdk1 inhibitor purvalanol A (Fig. 1E), which showed identical results as those obtained in the RNAi studies. Also, the diminished Cdk1 activity in the cells lacking Survivin, and therefore, the impossibility of a downstream prometaphase blockage was reaffirmed by comparing the activity of the mitotic kinase in the absence of Survivin and the Cdk1 activity in a real prometaphase blockage triggered by the microtubule-depolymerizing agent nocodazole (28) (Fig. 1G).

The results obtained with asynchronous cultures could be reproduced with synchronous cells. Here, a much more robust mitotic blockage, and low Cdk1 activity was observed (Figs. 2A–C). From this data, I inferred that the mitotic cells that accumulated in the G2/M-phase peak in the absence of Survivin had to be in either G2 or early prophase, as their asynchronous counterparts, since if there had been a large spill of G2/M-phase into G1, as a result of aborted mitosis, the majority of these cells should have contributed to increasing the *>4N* peak, which was not the case, due to the fact that HeLa cells possess very low levels of the p53 tumor suppressor (41), and therefore lack an effective G1 checkpoint (42). The role of Survivin in mitotic progression was further proved by the partial override of the G2/M-phase blockage (this partial rescue was probably due to the still presence of the Survivin siRNA in these cells) when this protein was reintroduced into the cells from which it was previously ablated.

The use of the Survivin double mutant provided a precise timeline of the mitotic defects that occur when Survivin’s function is impaired in cells about to enter mitosis. In effect, the results in this paper now show that Survivin is first needed to commit to mitosis (i.e. round), and that in its absence or malfunction, cells block at G2/prophase (Fig. 5D, *bottom*), a result also suggested by the RNAi data. Since both these Survivin antagonists prevent Cdk1 activation (Figs. 1F and 5B), and this enzyme’s activity is needed to initiate mitosis, it is logical to think that the early blockage caused by these two reagents might have been due to the impossibility for the transfected cells to mount a high enough Cdk1 activity with which transverse the G2/M-phase threshold. Second, the Survivin double mutant experiments also showed that in the absence of a functional Survivin protein, and subsequent low Cdk1 activity, some cells can still commit to mitosis (33%, see Fig. 5D, *bottom*), however in most cases stall or die at this stage. Interestingly, similar results were obtained by other authors in human cells when interfering with their Cdk1 levels via RNAi (43). In this case, 20% of cells managed to enter mitosis, however, these Cdk1-attenuated cells appeared to be impaired in their ability to phosphorylate their mitotic targets (43). From these results, it is possible to conclude that one other actor, which might be responsible for the stall of cells at prometaphase following Survivin abrogation and override of the G2/M-phase checkpoint could be Cdk1 in its ability to regulate the CPC and spindle checkpoint (1, 44, 45, 46). Finally, in the absence of Survivin only a few cells would manage to correctly enter anaphase, and would generally proceed into cell death or endoreplication.

### Centrosomal Cdk1 Does Not Accumulate in the Absence of Survivin and this May Cause a Short-Circuit between this Kinase and its Cdc25B Activator

Cdk1 is activated by the dual-specificity phosphatase Cdc25 (5), with isoform B being the first one that acts on Cdk1 at the centrosome during early prophase (6, 7), and isoforms A and C being subsequently active at the cytosol and nucleus in a process that amplifies the initial kinase activity (5), and commits cells to mitosis (13, 14). The data in this article now shows that when the Survivin function was compromised in HeLa cells that had not entered mitosis yet, these samples arrested at early prophase (see above discussion). From this data, I inferred that this abnormal phenotype might be the result of a short-circuit in the Cdc25B-Cdk1 axis activation. In agreement with this idea, centrosomal Cdk1 was greatly reduced in the Survivin-depleted samples (Fig. 6B and C). However, still some Cdk1 activity could be seen at early time points in cytosolic extracts from Survivin-depleted cells that I attribute to the initial Cdk1-Cyclin A complex, which escapes Wee1/Myt1-inhibitory phosphorylation, and is required to activate Aurora A (47), itself the activator of Cdc25B at the centrosome (48).

The immunofluorescence studies showed a decrease in the amount of Cdk1 at the centrosomes of Survivin-depleted cells, and a reduction in the number of cells with separated spindle poles, a phenotype previously ascribed to Cdk1 impairment (20, 21). From these results, I postulate that Survivin probably acts as some kind of mediator that facilitates the activation of Cdk1 via Cdc25B at the centrosome, and that this is the reason why in its absence, cells stall at early prophase.

### Survivin Activates the Cdk1 Kinase Via Phosphatase Cdc25B

In agreement with Survivin depletion probably affecting the activation of Cdk1 via Cdc25B at the centrosome, I could detect the accumulation of a phosphorylated Cdk1 form previously ascribed to the inactive Cdk1 (Fig. 7A). Also, a Cdk1 protein slowly migrating in SDS-PAGE (Fig. 7B), which was previously determined to be inactive (27, 35), could be seen in the Survivin-depleted cells. Interestingly, the shift between the inactive and active Cdk1 forms could be induced in a cell-free assay where Survivin was depleted from mitotic lysates, and then added back (Fig. 7C), and also replicated in a dose-dependent manner using interphase extracts (Fig. 7D) that phosphorylated Histone H1 (Fig. 7E). This latter result might point at an unknown function of Survivin at interphase, which could be behind the cell death observed in cells expressing the Survivin double mutant while progressing towards the G2/M-phase checkpoint (Fig. 5D, *bottom*).

Initially, the role of Survivin as a bridge between Cdc25B and Cdk1 was further supported by the interaction of the former protein with the latter two *in vitro* (Figs. S2B and 8A). However, when I tried to find a Cdc25B-Cdk1 complex in cell lysates, which also contained centrosomes, of HeLa cells treated with the control siRNA, I did not get the expected result, and counterintuitively, I could only see a Cdc25B-Cdk1-Cyclin B1 interaction in the Survivin-depleted samples (Fig. 8B), which according to the centrosomal data, had to be cytosolic. Taken into account the Histone H1 phosphorylation data, the Cdc25B bound to Cdk1 had to be inactive, and indeed, when I looked at the immunoprecipitated Cdc25B-Cdk1-Cyclin B1 complexes in detail, I could see the slow-migrating Cdk1 band previously ascribed to the inactive kinase (27, 35). This conclusion was corroborated by directly measuring the Cdc25B activity in samples where Survivin was ablated (Figs. 8E and F). From these results, I discarded the role of Survivin as a linker between Cdc25B and Cdk1. An alternative here might be that Survivin acts as some kind of centrosomal anchor or scaffold for the inactive Cdc25B-Cdk1-Cyclin B1 complex, which by this means could come close to its centrosomal activator/s.

In the absence of Survivin, Cdc25B accumulated to a great extent (Figs. 8B, C, D and F), and remained in complex with Cdk1 for an extended time (Figs. 8B and F). This was very different to the previously reported short stability of this phosphatase (49). Moreover, Cdc25B normally accumulates at G2, and its activity precedes Cdk1 activity, followed by both events rapidly declining after G2/M-phase (49, 50, 51), a result that I could reproduce in my studies in the control cells (Fig. 8F), as a consequence of Cdk1-dependent proteasomal degradation (52).

Overexpression of Cdc25B normally induces mitotic entry (39, 53). However, when the exogenous Cdc25B phosphatase was expressed in Survivin-depleted G2/M-phase blocked HeLa cells, this protein could not rescue the blockage (Fig. 9A, *right*, lower right graph), confirming that Survivin is required to activate Cdc25B. In agreement with this theory, when a gain-of-function Cdc25B mutant was expressed in G2/M-phase cells lacking Survivin, they managed to enter mitosis (Fig. 9B), supporting a role for Survivin in signaling through the Cdc25B-Cdk1 axis.

### Early Prophase Physiognomy and Reversibility

Following the accumulation of data in this project, it was clear that the Survivin protein is necessary to assemble a functional Cdk1 complex in early prophase. This was a valid conclusion except for the fact that only a small number of rounded cells were observed following their treatment with different Survivin antagonists (Figs. 1C and 5D, *bottom*). Searching through the literature to find an explanation for this observation, I discovered that early prophase cells do not always look round, but on the contrary, many times remain spread, resembling the physiognomy of interphase or G2 cells (13, 14). The choice between these two different appearances seems to rely on the amount of Cdk1 activity in the nucleus. Accordingly, it was quite possible that many of the cells that looked like being in G2, or interphase, following interference with the Survivin function, were actually in early prophase.

Early prophase is also a reversible phase, and this reversibility can be triggered by multiple insults. Antephase is the stage to which cells resort following the activation of a G2/M-phase checkpoint branch that responds to stress insults, and is mediated by p38 (54). This checkpoint is different to the one triggered by DNA damage, which depends on ATM and/or ATR activity (55). I would speculate here that many of the cells that entered mitosis with a low Cdk1 activity, as a result of Survivin interference, might have activated their antephase rather than their DNA damage checkpoint, and as a consequence returned to G2, hoping to re-enter mitosis at a better time. The antephase checkpoint only works while the activity of Cdk1 is not very high, or before nucleolar breakdown (54). This would explain why cells that were treated with the Survivin peptide could not return to G2, and continued into the apoptotic program (see below).

### Interference with Committed Mitotic Cells Via a Cdk1-Binding Survivin Peptide Results in Apoptosis

The Survivin peptide was made with the intention to interfere with the assembly of the Survivin-Cdk1 complex, which accumulates at mitosis. In agreement with this, treatment of HeLa cell lysates from nocodazole-treated HeLa cells with the Survivin peptide caused complex disassembly, and loss of Cdk1 activity (Fig. S4B), proving that Survivin needs to be bound to Cdk1 for the kinase to be active. Also, treatment of HeLa cells with the Survivin peptide correlated with several spindle abnormalities, including the *mini spindle* phenotype that inspired this work, in committed mitotic cells, as judged by their attempt at forming a DNA segregation apparatus (5). Interference with Cdk1 activity in cells that have disassembled their nuclear envelope (i.e. committed cells) (8), and subsequently progressed to prometaphase with a fully active Cdk1 kinase (13, 14) might lead to conflicting orders and mitotic disarray (Fig. 3), as Cdk1 is needed for spindle formation at this stage (20, 21, 56, 57). Another consequence of interfering with Cdk1 activity in committed mitotic cells might be *mitotic abortion* and cell death (15). This is precisely what happened in HeLa cells treated with the Survivin peptide. Here, I want to insist on the fact that the cell death phenotype was not observed in the Survivin siRNA- or Survivin double mutant-treated samples, and that this might have to do with these cells having enough time to find refuge at the G2/M-phase checkpoint following the Survivin antagonist treatment. In support of the vulnerability of tumoral cells committed to mitosis and targeted with a Cdk1 inhibitor, cancer cells and tumors in a cancer xenograft model were very sensitive to a combination of taxol and purvalanol A but not to the reverse order of the treatment (58), indicating that when challenged cancer cells are allowed to rest at the G2/M-phase checkpoint, they can avoid cell death.

Regarding the cell death observed when HeLa cells were treated with the Survivin peptide, it has been shown that Cdk1-Cyclin B1 activity is required to phosphorylate procaspase 8 (59) and procaspase 9 (60), and subsequently block the extrinsic (59) and intrinsic (60) apoptotic pathways in cancer cell lines. Here, blockage of Cdk1 activity by Cyclin B1 RNAi or the Cdk1 inhibitor RO-3306 (59), or short-circuit of the kinase by the expression of non-phosphorylatable caspase proforms (59, 60), led to apoptosis. Similarly, when I treated HeLa cells with the Survivin peptide, I could detect caspase 8 and 9 activity triggered by this reagent (see Figs. 4C and D). Undoubtedly, from these results, the Cdk1 kinase rises as some kind of mitotic protective shield but also as a factor, which might contribute to tumorigenesis if deregulated. Curiously, these have been roles traditionally assigned to Survivin, and we may have now identified its partner responsible for these phenotypes.

There has been a lot of speculation regarding the two seemingly roles of Survivin in cancer regulation, namely: the one in mitosis and the one in apoptosis (for reviews on these two different views see 61 and 62). In this respect, I propose here an integrating theory in which the two roles of Survivin can coexist. In this model, Survivin’s main function in cancer cells would be controlling mitotic entry through its regulation of Cdk1 activity. This role of Survivin would have downstream repercussions on the CPC function, and ultimately would lead to apoptosis (18) when the Cdk1 is deregulated through interference with Survivin at the checkpoint via biological or chemical insults.

### Survivin Binds to the αC/β4 Loop in Cdk1, a Region Involved in Protein-Protein Interactions, Binding of the Hsp90-Cdc37 Complex and Regulation of Kinases’ Activity

The Survivin peptide spanning the Cdk1-binding region (Figs. S3A and C) comprises several negatively-charged residues that reside on the Survivin dimer’s acidic surface (32). In fact, even though I used the SUR D70A/D71A mutant as a Survivin antagonist, both the GST-SUR 71–142 truncated protein, which could not bind the kinase (Fig. S2C), and the alanine point mutant SUR D71, which slightly did (Fig. S5, *bottom*), suggested that only the Asp70 residue is crucial for binding to Cdk1. From these observations, I predicted that Survivin should probably recognize a region in the Cdk1 kinase with a net positive charge, and indeed, I was able to narrow down the Survivin-binding sequence in the Cdk1 kinase to amino acids Lys56 through Met85, a region that includes the kinase’s αC/β4 loop, which contains several basic residues (Fig. S3B). Residues 66 through 85 were also part of the original Cdk1 sequence recognized by Survivin, however this kinase region harbors numerous hydrophobic amino acids, and therefore I found it unlikely to be part of the Survivin-binding domain.

The αC/β4 loop is a conserved structural motif present in all eukaryotic protein kinases, which bridges the αC helix in the N lobe and regions at the top of the C lobe (63). In most kinases, this loop spans 8 amino acids, which follow the consensus sequence: L-x-H-P-N-T-V-x, where x represents any amino acid (64). Functionally, it has been hypothesized that the αC-β4 loop may act as a molecular brake for protein kinases by maintaining auto-inhibitory interactions via hydrogen bonding with the hinge region in the C lobe (65, 66, 67). In support of this theory, many mutations have been found at this location, which confer constitutive kinase activity and/or drug resistance, and play a direct role in cancer progression (64). The αC-β4 loop is also a site for protein–protein interactions, and as such, it recognizes the molecular chaperone Hsp90 and its co-chaperone cdc37 (68, 69), proteins that together promote the proper folding of 60% of the human kinome (69, 70). By cryoEM structure of an Hsp90-Cdc37-Cdk4 complex, a general mechanism of Hsp90-Cdc37 action has been postulated (71), where a conserved H-P-N motif in Cdc37 would mimic the turn within the kinase αC-β4 loop, and push against the kinase αE helix in the C-lobe, preventing the contact between the kinase N and C lobes. As a result of this intermolecular interaction, the kinase would be initially stabilized, and would expose other motifs to which Hsp90 could bind. From all this information, it is tempting to speculate that Survivin might also have a role in the regulation of Cdk1 inter-lobe movement and its activation. In support of this view, Survivin recapitulates some of Cdc37’s features. In effect, apart from binding to the αC-β4 loop region as already stated, Survivin, like Cdc37, binds to the N-terminus of the Hsp90 chaperone (72, 73). Also, I could detect a complex between Survivin, Hsp90 and the active Cdk1 protein form at mitosis (Fig. S6). For these reasons, it is plausible to believe that coinciding with mitotic onset, Survivin might function as some kind of Hsp90 co-chaperone. Alternatively, Survivin might bind the chaperone machinery, once the Cdk1 complex has been assembled, and bring it close to its activator/s at the centrosome. Still, a third possibility might be that Survivin unloads the Hsp90-assembled Cdk1 complexes, and delivers them to its centrosomal activator/s. In this regard, this work showed that when Survivin was absent, an inactive cytosolic Cdc25B-Cdk1-Cyclin B1 complex accumulated, and here, it would be interesting to find out whether this complex was bound to Hsp90. A function of Survivin in mediating Cdk1 through Hsp90 is however a bit controversial, as it encounters those who claim that Cdk1 is not a Hsp90 client (70), and others that support the opposite view (69, 74, 75).

## CONCLUSIONS

Survivin has been called a *cancer gene* (76). This paper now shows that this title may have in part been earned due to an up-to-date unreported role of Survivin in the activation of the Cdc25B-Cdk1 complex. In this regard, there is plenty of research showing a correlation between Cdc25B signaling and tumorigenesis, and it would make perfect sense that cancer cells hijack this pathway in order to control mitotic entry. The connection between Cdc25B and cancer has led to an overwhelming effort trying to develop efficient Cdc25B inhibitors (77). These inhibitors however most of the time target the Cdc25B catalytic domain, and here, an exciting alternative might be finding small molecules that disrupt the Survivin-Cdc25B interaction.

In this work, I have also shown that the absence of Survivin leads to an early prophase blockage due to low Cdk1 activity, and propose that this phenotype is very sensitive to events, which override the G2/M-phase checkpoint. Therefore, a combination of drugs that promote mitotic entry, and then inhibit Cdk1 activity might be a very effective way to combat cancer.

Allosteric inhibitors are also on the way that bind to the αC-β4 loop in Cdks (78), and this paper reinforces the importance of this site by proving that Survivin is another protein that binds to this epitope. From different studies, the αC-β4 loop is now rising as some sort of a hub through which kinases communicate with other proteins, and signals can be sent through a myriad of pathways. Therefore, elucidating the exact function of this region in kinases, and the role of the chaperone machinery at this location, may help refine the design of new drugs against cancer.

To end, I would like to reiterate the connection between Cdk1 activity and CPC’s regulation, and the possible role of Survivin at multitasking between kinases. In this context, Survivin might operate as a coordinator of upstream and downstream mitotic events, which would lead to smooth progression through mitosis.

## MATERIALS AND METHODS

### Plasmids, Cloning, Recombinant Proteins and Peptides

Full-length human cDNAs encoding Cdk1 (BC014563) and Cdc25B (BC051711) were obtained from Open Biosystems. Cdk1 and Cdc25B cDNAs were subcloned in frame with the N-terminal His-tag into pRSETa (Novagen, Merck Biosciences). Cdc25B cDNA was also subcloned into pcDNA3 without (pCdc25B). pGST-Survivin (plasmid containing a Survivin sequence cloned in frame with the N-terminal GST-tag into pGEX (Pharmacia)) and pGFP-Survivin (plasmid made in pcDNA3 including an N-terminal GFP sequence (pGFP)) were available in the laboratory. Deletion mutants for His-tagged Cdk1 (residues 1–43, 1–56, 1–85, 1–183, 9–85, 36–85 and 182–297), Cdc25B (residues 391–580 (ΔNCdc25B)) and GST-Survivin (residues 1–70, 15–142, 38–142, 55–142, 71–142 and 81–142) were generated by PCR. Alanine mutagenesis of the Survivin sequence (pGST-Survivin) was carried out using the QuikChange^R^ Site-Directed Mutagenesis Kit (Stratagene). Plasmid encoding GFP-Survivin D70A/D71A (pGFP-Survivin D70A/D71A) was made by cloning the Survivin D70A/D71A sequence obtained by alanine mutagenesis into pGFP. All constructs were confirmed by DNA sequencing. His-tagged and GST-fusion proteins were expressed in BL21 *E. Coli* strain, and isolated by affinity chromatography on Ni^2+^-charged agarose (Novagen, Merck Biosciences), or glutathione agarose (Sigma-Aldrich). A peptide comprising residues Ala55 throughout Asp70 in Survivin (AQCFFCFKELEGWEPD), and a scrambled version of the same peptide (EPCWDECFAEKFQGFL), both containing a Biotin moiety and a HIV tat cell-permeable sequence at the N-terminus, were synthesized using f-BOC chemistry, purified by HPLC and analyzed by mass spectrometry (Peptron, Inc., Daejeon, South Korea or W. M. Keck Biotechnology Research Center, Yale University, School of Medicine).

### Cell Culture, Synchronization and Transfections

Cervical carcinoma HeLa cells (ATCC, Manassas, VA) were maintained in culture as described (16). Cells were synchronized by incubation with 2 mM thymidine (G1/S-phase) or 10 μM nocodazole (M-phase). In some experiments, cells were treated with the Cdk1 inhibitor purvalanol A (10–20 μM) for 16 h. On other occasions, mitotic cells were prepared by shaking off the plates, or collecting the samples 9 to 14 h post thymidine release. Gene targeting by small interfering RNA (siRNA) was carried out with a control or a Survivin-siRNA oligonucleotide characterized in earlier studies (16). When using synchronized cultures, cells were first treated with the appropriate siRNA, and then incubated with thymidine for 48 h. Cells were then released into fresh medium, and samples taken at increasing time intervals. Transfection of adenovirus vectors encoding GFP-Survivin (pAd-Survivin) or GFP (pAd-GFP) was performed as indicated (79). For plasmid transfection, cells (50–70% confluency) were incubated with 1–2 μg of plasmid in 1 mL Optimem-I and 4 μL lipofectamine/well for 4 h. When siRNA and pAd vectors were co-transfected, cells were first incubated with control or Survivin siRNA, and synchronized in G1/S-phase for 48 h. Cells were then released into fresh medium, transfected with the appropriate pAd vector, and harvested at increasing time intervals. In the case of transfections with siRNA and Cdc25B plasmids, cells remained in thymidine for 32 h after the siRNA treatment, plasmids were transfected in the presence of thymidine for 4 h, and cells were finally released 12 h post-transfection and harvested as indicated.

### Preparation of Cell Lysates and Isolation of Centrosomes

Cells were lysed in 1 mM HEPES, pH 7.2, 0.5% IGEPAL CA-630, 0.5 mM MgCl_2_, 0.1% β-mercaptoethanol, 50 mM NaF, 1 mM Na_3_VO_4_ plus protease inhibitors for 30 min on ice. The cell lysate was pipetted up and down five times, centrifuged at 2,500 g for 10 min, and supernatant was adjusted to 10 mM HEPES, pH 7.2 final concentration. For isolation of crude centrosomes, 1 μg/mL DNAse I was added to the lysate, and incubation proceeded for 30 min on ice. The mixture was then overlaid on a 60% sucrose cushion, containing 10 mM HEPES, pH 7.2, 0.1% Triton-X100 and 0.1% β-mercaptoethanol, and centrifuged at 10,000 g for 30 min. The interface of the sucrose cushion containing enriched centrosomes was collected for the various experiments. Protein concentration was estimated by the BCA assay (Pierce).

### GST-Pull Downs, Immunoprecipitations and Antibodies

Pull-down experiments with His-tagged proteins mixed with GST, GST-Survivin or GST-Survivin mutants were carried out as described (78). For streptavidin-binding experiments, 50 μg of biotinylated peptides were bound to 25–50 μl of a 50:50 slurry of streptavidin agarose (Sigma-Aldrich), mixed with recombinant proteins or cell lysates, and analyzed by Western blotting. Immunoprecipitation using primary antibodies (10 μg/mL), and 200–400 μg of total or fractionated cell extracts were carried out as described (80). The following antibodies to Cdk1, PT14/PY15-Cdk1, 14–3-3 β, Lamin B (all from Santa Cruz Biotechnology), Cyclin B1 (PharMingen), Survivin (NOVUS Biologicals), α-tubulin (clone B-5–1-2) (Sigma-Aldrich) and His-tag (Invitrogen) were used. Protein bands were visualized by enhanced chemiluminescence (Amersham Biosciences).

### Analysis of Cdk1 Activity

Cell lysates in 10 mM HEPES, pH 7.2, 0.5% IGEPAL CA-630, 0.5 mM MgCl_2_, 0.1% β-mercaptoethanol, 50 mM NaF, 1 mM Na_3_VO_4_ plus protease inhibitors were used to immunoprecipitate Cdk1. Kinase activity was assayed as described (22). For the *in vitro* Cdk1 activation experiments, synchronized HeLa cell lysates were prepared by incubation for 30 min on ice in 10 μM HEPES, pH 7.4, 10 μM KCl, 1.5 mM MgCl_2_ and 2 μM DTT plus protease inhibitors, and passing the cells ten times through a 27 ½ G needle. Lysates were centrifuged at 2,500 g for 10 min, and supernatants were adjusted to 10 mM HEPES, pH 7.4, 100 μM KCl and 1 mM DTT. Survivin was depleted in the lysates by rotation with 25–50 μl of a 50:50 slurry of protein A agarose (Boehringer Ingelheim) bound to Survivin antibody overnight at 4°C. Survivin-depleted supernatants were supplemented with an ATP-regenerating system (2 mM ATP, 10 mM phosphocreatine and 3.5 U creatine kinase per 100 μL lysate), and incubated with 0.5–3 μM GST or GST-Survivin for 1 h at 30°C. For the peptide interference of Cdk1 activity *in vitro*, lysates were prepared from nocodazole-treated cells as described for the Cdk1 activation experiments but this time the endogenous Survivin protein was not depleted, and samples were incubated with 1–5 μM peptides for 1 h at 30°C. All reactions were terminated by a 10-fold dilution in a buffer containing 50 mM NaF and 1 mM Na_3_VO_4_. Phosphorylated Cdk1 proteins were immunoprecipitated, resolved by SDS-PAGE and identified by Western blotting. Cdk1 activity was assayed as described (22).

### Cdc25B Activity Assay

Cdc25B protein was immunoprecipitated from cell lysates as described (80), and phosphatase activity was assayed by 3-O-methyl fluorescein phosphate (OMFP) hydrolysis at 30°C in assay buffer (100 mM Tris, pH 8.2, 40 mM NaCl, 1 mM DTT and 20% glycerol) (38). Fluorometric detection was carried out at 485 nm excitation and 530 nm emission wavelengths (Photon Technology International, Inc.). Fluorescence slopes were calculated and normalized against values obtained with control, non-binding IgG.

### FACS Analysis

DNA content in asynchronous or thymidine-synchronized HeLa cell cultures was determined by FACS analysis (propidium iodide staining and flow cytometry) as described (22). Data were quantified using the FlowJo cell cycle analysis software.

### Analysis of Apoptosis

DNA integrity was analyzed by scoring the sub-G1 population (*<2N*) in FACS analysis. Caspase activity was detected by incubating siRNA transfected HeLa cells with a fluorescein-conjugated caspase inhibitor (FAM-DEVD-FMK). Alternatively, HeLa cell lysates were analyzed by Western blotting using an antibody to procaspase 3.

### Fluorescence Microscopy

Transfected HeLa cells seeded onto optical grade glass coverslips were fixed in ice-cold 4% paraformaldehyde/PBS, pH 7.2 for 1 h, washed and permeabilized in 0.2% Triton-X100/PBS, pH 7.2 for 30 min at 22°C, except when they were transfected with GFP constructs, in which case, cells were directly analyzed under the microscope. Coverslips were blocked in 3% BSA/0.1 *%* Tween-20/PBS, pH 7.2 for 30 min at 22°C, and incubated with primary antibodies appropriately diluted in blocking buffer for 1 h at 22°C. Cells were incubated with Alexa Fluor (Invitrogen) secondary antibodies for 1 h at 22°C in the dark. When cells were transfected with biotinylated peptides, they were directly incubated with Texas red-streptavidin, following fixation and permeabilization. DNA was stained with 10 μg/mL DAPI (Sigma-Aldrich). Coverslips were analyzed using an inverted fluorescence microscope (Olympus IX71). Pictures were taken using a microscope-attached camera (Nikon), and data was computer-analyzed by the IPLab software.

### Time-Lapse Video Microscopy Analysis

Thymidine synchronized HeLa cells seeded onto Lab-Tek two-chambered borosilicate coverglass slides (Nalge Nunc International) were transfected with the appropriate GFP plasmid, released into fresh medium, and imaged using an inverted microscope (Zeiss) with a 20X numerical aperture 0.5 objective lens, a spinning-disk confocal scan head (Perkin Elmer Life and Analytical Sciences) and an Orca-ER cooled CCD camera (Hamamatsu) under conditions of CO_2_ exchange (0.5 L/min) at 37°C. Time-lapse imaging was conducted using a multidimensional image software (Metamorph 6.3r6) to acquire images every 10 min for 24 h, observe multiple x,y-coordinates at every time point and acquire six optical sections over a 10 μm z-series (2 μm step size) at each coordinate. A z-stack of GFP images was taken at the start and end of each time-lapse experiment.
